# National suicide management guidelines recommending family-based prevention, intervention and postvention and their association with suicide mortality rates: systematic review

**DOI:** 10.1192/bjo.2022.15

**Published:** 2022-02-24

**Authors:** Balpreet Panesar, Divya Soni, Mohammed I. Khan, Faris Bdair, Matthew Holek, Talha Tahir, Julia Woo, Nitika Sanger, Nonhlanhla P. Khumalo, Luciano Minuzzi, Lehana Thabane, Zainab Samaan

**Affiliations:** Neuroscience Graduate Program, McMaster University, Hamilton, Ontario, Canada; Faculty of Medicine, University of Toronto, Ontario, Canada; Biostatistics Unit, St Joseph's Healthcare, Hamilton, Ontario, Canada; Mathematical and Computational Science Undergraduate Program, Stanford University, California, USA; Health Sciences Undergraduate Program, McMaster University, Hamilton, Ontario, Canada; Michael G. DeGroote School of Medicine, McMaster University, Hamilton, Ontario, Canada; Department of Psychiatry and Behavioural Neurosciences, McMaster University, Hamilton, Ontario, Canada; Medical Science Graduate Program, McMaster University, Hamilton, Ontario, Canada; Division of Dermatology, Department of Medicine, Groote Schuur Hospital, and University of Cape Town, South Africa; Department of Psychiatry and Behavioural Neurosciences, McMaster University, Hamilton, Ontario, Canada; Department of Health Research Methods, Evidence and Impact, McMaster University, Hamilton, Ontario, Canada; and Centre for Evaluation of Medicines, Programs for Assessment of Technology in Health (PATH) Research Institute, McMaster University, Hamilton, Ontario, Canada; Department of Psychiatry and Behavioural Neurosciences, McMaster University, Hamilton, Ontario, Canada; and Department of Health Research Methods, Evidence and Impact, McMaster University, Hamilton, Ontario, Canada

**Keywords:** Suicide, national guidelines, family, systematic review, recommendations

## Abstract

**Background:**

Suicidal behaviour remains a major public health concern and countries have responded by authoring guidelines to help mitigate death by suicide. Guidelines can include family-based recommendations, but evidence for the level and category of family-based involvement that is needed to effectively prevent suicide is unclear.

**Aims:**

To explore the association between family-based recommendations in guidelines and countries’ crude suicide rates. PROSPERO registration: CRD42019130195.

**Method:**

MEDLINE, Embase, PsycInfo, Web of Science and WHO MiNDbank databases and grey literature were searched within the past 20 years (1 January 2000 to 22 June 2020) for national guidelines giving family-based recommendations in any of three categories (prevention, intervention and postvention).

**Results:**

We included 63 guidelines from 46 countries. All identified guidelines included at least one family-based recommendation. There were no statistically significant differences seen between mean World Health Organization crude suicide rates for countries that included only one, two or all three categories of family-based recommendations. However, a lower spread of crude suicide rates was seen when guideline recommendations included all three categories (mean crude suicide rates for one category: 11.09 (s.d. = 5.71); for two categories: 13.42 (s.d. = 7.76); for three categories: 10.68 (s.d. = 5.20); *P* = 0.478).

**Conclusions:**

Countries should work towards a comprehensive national suicide guideline that includes all categories of family-based recommendations. Countries with previously established guidelines should work towards the inclusion of evidence-based recommendations that have clear implementation plans to potentially help lower suicide rates.

Suicidal behaviour is the cause of death for close to 800 000 people worldwide every year and it remains a public health challenge for countries of various socioeconomic status.^1,2^ Many suicide interventions exist, but to allocate national spending to the most effective recommendations, it is important to identify what factors of effective recommendations help in reducing death by suicide.^3^

National suicide guidelines present country-specific prevention (recommendations employed during low to moderate risk of suicide), intervention (recommendations employed during high risk of suicide) and postvention (recommendations employed after completed suicide)^4^ recommendations. They are often accompanied by action plans, agendas and timelines that explain measures for implementation of the proposed recommendations.^5^ The World Health Organization (WHO) has stated that national recommendations are essential to put suicide risk onto political agendas, as the change-makers who can propose developmental and multisectoral recommendations are most often governments and political parties.^5^ Thus, it is important for countries to lay out evidence-based, collaborative recommendations to mitigate suicide risk.

National suicide guidelines formulate recommendations based on country-specific data on suicide and can include many kinds of recommendations, including family-based recommendations to assist efforts to mitigate risk of suicide. Many countries have conducted studies that have identified family to be a protective factor against suicide,^6–8^ where family cohesion, connectedness and openness support positive mental well-being of both adolescents and adults. Many studies have also identified family-related risk factors for suicide, for example family history of suicide and family dysfunction such as neglect and abuse.^9–11^ Family-based recommendations are directly related to the level of social support an individual receives.^7,8^ Since a high level of social support is a well-researched protective factor against suicide, looking at the inclusion of family-based recommendations will ultimately provide insight into the quality and depth of protection that national suicide guidelines are providing to individuals.

Overall, despite the identification of these family-based risk and protective factors as extensions of the level of social support in the literature, it is difficult to determine the level and kind of family involvement that is needed to mitigate suicide risk.^9^ Thus, to add to the literature investigating the effectiveness of national suicide recommendations, it is important to carry out a review of the inclusion of family-based recommendations in these guidelines, as countries may have taken liberties when translating identified family factors for suicide into suicide mitigation recommendations.

## Rationale

To help guide future family-based prevention, intervention and postvention recommendations, we identified and compared the type, number and strategy of national suicide guidelines that include family-based recommendations. We also compared the national rates of suicide deaths with the presence of family-based guidelines. Study findings will identify the categories of family-based recommendations included in guidelines and identify gaps and missed opportunities in the included recommendations, thus justifying a systematic search of evidence.

## Objectives

The question this review is examining is: in countries with national suicide guidelines published within the past 20 years, is the inclusion of family-based recommendations associated with a reduction in suicide rate?

The review aims to:

- assess whether the inclusion of families in the national guideline recommendations on managing suicidal behaviour is associated with reduction in the rate of death by suicide
- based on this assessment, suggest family-based recommendations to manage suicide risk.

## Method

### Protocol and registration

This systematic review was conducted to investigate the association between inclusion of families and family-based interventions in national suicide guidelines and reduction in the rate of death by suicide. The review was written using the Preferred Reporting Items for Systematic Review and Meta-Analysis (PRISMA) guidelines^12^ and a PRISMA checklist was completed. The complete protocol for this review has been published in a peer-reviewed journal and is registered with PROSPERO CRD42019130195.^13^ To execute a more comprehensive search strategy, the time frame for this review was changed from 10 year to 20 years.

### Eligibility criteria

Records were selected if they were national guidelines proposing suicide prevention recommendations or action plans. Any records published before the year 2000 were not included. If a country had published more than one guideline in the past 20 years addressing the same population, the most recent guideline was included. Guidelines that addressed different target populations within the same country were included. Countries whose suicide prevention plan is contained within national mental health guidelines and who did not have a separate national suicide guideline were not included. This is because our search strategy aimed to identify specific and thorough suicide guidelines aimed at the general population and therefore inclusion of national mental health guidelines that might include specific strategies to address mental health in addition to suicide would be outside the scope of this review. We did not include any research studies carried out on the national guidelines, as this review is examining the guidelines themselves. We did not limit by language, age, gender or country. Any guidelines that were not in English were translated by a native speaker of that language. Google Translate services were used if a native speaker could not be found (Google, Mountain View, US; see https://translate.google.com/).

### Information sources and search strategy

The search strategy was developed for Embase, MEDLINE, PsycInfo and Web of Science by an experienced health science librarian. It was broad and contained the search terms suicide, guidelines and consensus development. The full search strategy is available in the published protocol,^13^ but we have provided the strategy for MEDLINE in supplementary Table 9, available at https://dx.doi.org/10.1192/bjo.2022.15. We also searched the WHO MiNDbank (https://www.mindbank.info/), which includes suicide prevention strategies for 41 countries (e.g.^14^). We searched grey literature, including the National Guideline Clearinghouse, to account for any guidelines that were not peer reviewed.^15^ All the databases were searched from 1 January 2000 to 22 June 2020, to include recently published guidelines.

### Selection process

Using the established selection criteria, three pairs of reviewers completed the title and abstract screening and full-text screening phases independently in duplicate. If there was a disagreement that the pair of reviewers was unable to resolve through discussion, it was resolved with the consultation of a third reviewer.

### Data collection and data items

A pilot-tested data extraction form was used to extract the relevant information from the national guidelines. This was done in duplicate. The extracted information included the country, target population (i.e. youth, adults, seniors), year of publication, journal and details of the guideline's recommendation for suicide prevention. More specifically, we extracted information on: whether the guideline included the recommendation of family involvement, social support and any other support, and whether there are any data on uptake of recommendations (i.e. implementation of recommendations, effectiveness of recommendations). We also extracted the suicide rate for each country with guidelines and extracted the crude suicide rate as reported by the WHO.^16^

### Risk of bias of individual studies and within studies

As this is a review examining national guidelines, we used the Appraisal of Guidelines for Research & Evaluation II (AGREE II) tool.^17^ This assesses the quality of the guidelines on the basis of 23 items over 6 domains: scope and purpose, stakeholder involvement, rigour of development, clarity of presentation, applicability and editorial independence. This appraisal was conducted in duplicate. Additionally, we had planned on using the Grading of Recommendations Assessment, Development and Evaluation (GRADE) criteria to assess the quality and strength of the evidence, but as we were only able to qualitatively report the results with no meta-analyses, we used the AGREE II evaluations instead.^17,18^ The AGREE II scores were calculated by domain, which involves summation of scores from both appraisers and presenting the sum as a percentage by scaling it out of the maximum possible score for that domain.^17^

### Effect measures and synthesis of results

All included guidelines were qualitatively summarised and compared. We examined the differences between countries with guidelines that included family-oriented recommendations and those that did not. Recommendations were grouped manually into prevention (recommendations employed during low to moderate risk of suicide), intervention (recommendations employed during high risk of suicide) and postvention (recommendations employed after completed suicide) categories.^4^ SPSS Version 25.0 for Macintosh (IBM Corp, Armonk, US; see https://www.ibm.com/support/pages/downloading-ibm-spss-statistics-25) was used to generate descriptive box plots of the WHO crude suicide rates^16^ by the total number of family-based recommendations, the category of the recommendation (either prevention, intervention or postvention) and the total number of categories included in each guideline. Microsoft Power BI version 2.100.261.0 fo Windows (Microsoft, Redmond, US; see https://powerbi.microsoft.com/en-us/) was used to provide visualisations of the categories the recommendations were grouped into through the generation of filled maps.

SPSS was also used to conduct independent *t*-tests to compare mean WHO crude suicide rates between countries that did and did not include each of the three prevention, intervention and postvention categories. For all tests, the alpha level of significance was set to α = 0.05 and Levene's test for equality of variance was used to determine whether the equal variance was assumed or not assumed. Subsequently, the corresponding two-tailed significance value was reported. All tests reported mean values and standard deviation, the *t*-statistic, the degrees of freedom, the two-tailed significance value, mean difference, standard error difference and the 95% confidence interval of the difference.

A one-way ANOVA was conducted to compare the mean crude suicide rates between countries with guidelines that included one, two or three categories of family-based recommendations. The test reported the *F*-value and the *P*-value.

### Categories of recommendations

The categories included in the review are family-based prevention and intervention recommendations for risk of death by suicide, suicide attempts, suicidal ideation or suicide-related behaviour, as well as postvention recommendations for those bereaved by suicide.

### Outcome measures

The outcome in this review is the crude suicide rate estimates and crude suicide rates for the respective countries for which a guideline has been identified, as most recently reported by the WHO in 2019.^16^ These rates were accessed on 6 January 2022. Crude suicide rates are defined as the number of completed suicides in a given year divided by the total population for a specific country.^16^

## Results

### Study selection

**Fig. 1:**
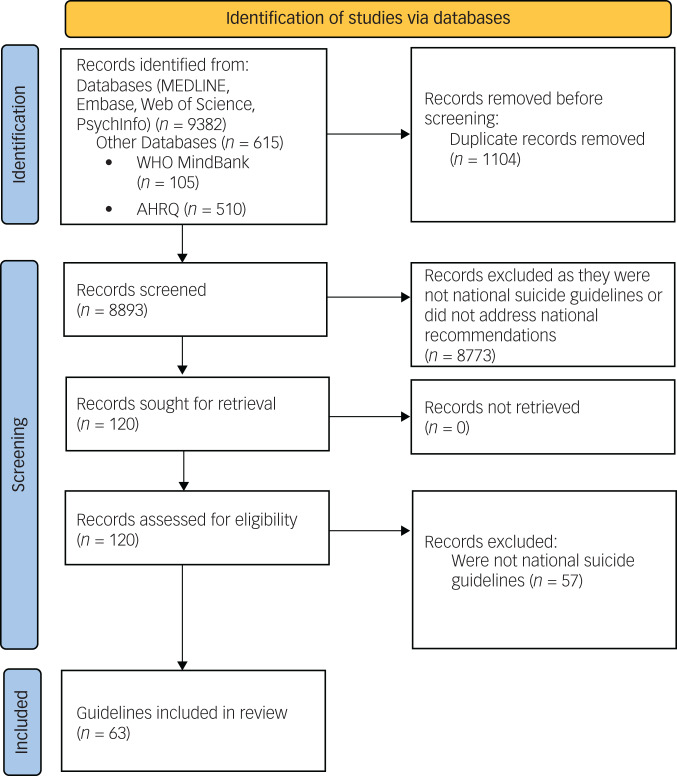
PRISMA flow diagram. AHRQ, Agency for Healthcare Research and Quality.

After removing duplicates, we identified a total of 63 guidelines and action plans from 46 countries. Of these, 52 were national suicide guidelines, 9 were national action plans or progress reports providing evidence for the implementation of the guidelines and 2 were international guidelines (Fig. 1). Action plans, progress reports and international guidelines were not considered in the quantitative analyses. A summary of the 52 national suicide guidelines can be found in Table 1.

**Table 1:** Total number of family-based recommendations in national suicide guidelines and crude suicide rates by country

Region	Year of Publication	Country	Family-based recommendations, *n*				WHO crude suicide rates per 100 000
			Prevention	Intervention	Postvention	Total	
Asia	2018	Afghanistan^22^	3	1	1	5	4.1
	2015	Bhutan^72^	7	0	1	8	4.6
	2017	Japan^63^	3	0	4	7	15.3
	2013	Malaysia^68^	0	5	2	7	5.7
	2018	South Korea^59^	3	0	1	4	28.6
	1997	Sri Lanka^28^	1	1	0	2	14
	2010	Uzbekistan^38^	4	0	1	5	8
Europe	2011	Austria^53^	1	0	0	1	14.6
	2015	Belarus^47^	1	1	0	2	21.2
	2012	Belgium^48^	2	0	1	3	18.3
	2013	Bulgaria^31^	1	0	0	1	9.7
	2011	Croatia^60^	3	0	0	3	16.4
	2006	Denmark^26^	0	3	1	4	10.7
	2012	^a^England^32^	2	0	5	7	7.9
	2020	Finland^27^	5	1	1	7	15.3
	2011	France^50^	0	1	1	2	13.8
	2015	Ireland^87^	3	0	2	5	9.6
	2017	Italy (adult penitentiary)^56^	4	0	0	4	6.7
	2017	Italy (youth penitentiary)^67^	4	0	0	4	6.7
	2017	Lithuania^39^	2	0	0	2	26.1
	2015	Luxembourg^40^	3	1	0	4	11.3
	2007	Netherlands^19^	3	0	2	5	11.8
	2019	^a^Northern Ireland^65^	2	0	5	7	7.9
	2020	Norway^54^	4	0	2	6	11.8
	2013	Portugal^73^	5	0	1	6	11.5
	2013	Scotland^33^	1	1	1	3	
	2006	Spain^25^	6	3	0	9	7.7
	2008	Sweden^41^	2	0	0	2	14.7
	2016	Switzerland^43^	2	0	1	3	14.5
Africa	2012	Namibia^84^	3	2	0	5	9.7
Oceania	2008	Australia^88^	5	0	1	6	12.5
	2013	Australia (aboriginal)^62^	3	0	2	5	12.5
	2019	New Zealand^44^	8	1	2	11	11
	2016	Cook Islands^52^	4	0	0	4	
	2015	Fiji^42^	4	0	0	4	9
South America	2015	Argentina^51^	2	0	1	3	8.4
	2017	Brazil ^45^	1	1	1	3	6.9
	2013	Chile^61^	0	3	1	4	9
	2015	Guyana^14^	2	0	2	4	40.3
	2016	Suriname^46^	1	0	3	4	25.4
	2011	Uruguay^36^	3	1	0	4	21.2
North America	2018	Canada^20^	5	0	0	5	11.8
	2016	Canada (National Inuit Strategy)^20^	3	0	0	3	11.8
	2017	Canada (armed forces)^20^	4	0	1	5	11.8
	2013	Canada (Aboriginal youth)^20^	3	0	0	3	11.8
	2010	Costa Rica^58^	3	0	0	3	8.1
	2014	Dominican Republic^55^	4	0	0	4	4.9
	2018	El Salvador^66^	6	0	0	6	6.1
	2000	Nicaragua^69^	0	7	1	8	4.4
	2006	Panama^23^	1	0	0	1	2.9
	2012	USA^48^	4	2	4	10	16.1
	2011	USA (American Indian) ^21^	1	0	0	1	16.1

WHO, World Health Organization.

a. The crude suicide rates for Northern Ireland and England are under the same category in the WHO database.

### Study characteristics

All 52 national suicide guidelines that were identified included some mention of family-based recommendations. Two guidelines did not include measures for implementation: these were The Netherlands guideline and the Canadian veteran guideline (Supplementary Table 1).^19,20^ Eight guidelines did not include measures of effectiveness: these were the Afghanistan, Sri Lanka, Denmark, Finland, Canadian aboriginal, Spain, Panama and USA aboriginal guidelines.^21–28^ A total of nine action plans were found during the systematic search. Specifically, Australia, Canada, Bulgaria, The Netherlands, England, Scotland, South Korea, Japan and Uruguay all had action plans or progress reports found outside of the national suicide guideline.^24,29–36^ More specifically, South Korea and Japan had an accompanying excerpt from parliament integrating the guidelines into judiciary efforts such as laws and acts.^34,35^ Bhutan,^37^ Uzbekistan,^38^ Lithuania,^39^ Luxembourg,^40^ Sweden^41^ and Fiji^42^ mentioned in their guidelines that an action plan existed or was in the process of being implemented. Switzerland,^43^ New Zealand,^44^ Brazil^45^ and Suriname^46^ formally contained action plans within the guideline documents. A total of 10 guidelines did not give a rationale for their inclusion of family-based recommendations. These were Belarus,^47^ Belgium,^48^ Bulgaria,^49^ France,^50^ Scotland,^33^ Sweden,^41^ Fiji,^42^ Argentina,^51^ Brazil^45^ and the Canadian Framework.^20^ Guidelines from the Cook Islands,^52^ US aboriginal guideline,^21^ Sri Lanka,^28^ Austria,^53^ Panama^23^ and Norway^54^ did not include rationale based on protective or risk factors for suicide, but included rationale based on other family- and suicide-related evidence. The Dominican Republic,^55^ Italy adult prison guideline,^56^ New Zealand,^44^ Uruguay^36^ and the USA^57^ included protective factors but lacked risk factors when providing rationale for including family-based recommendations in their guidelines. A total of 17 guidelines, including Costa Rica,^58^ Canadian aboriginal, veteran and youth guidelines^20^ South Korea,^59^ Croatia,^60^ England,^32^ Lithuania,^39^ Spain,^25^ Chile,^61^ Switzerland,^43^ Australian aboriginal guidelines,^62^ Afghanistan,^2^ El Salvador,^66^ Nicaragua,^69^ Japan^63^ and Guyana,^64^ included risk factors for suicidal behaviour but not protective factors in their listed rationale for including family-based recommendations. A total of 6 countries in South America, 7 from North America, 4 from Oceania, 1 from Africa, 7 from Asia and 21 from Europe had guidelines identified (supplementary Tables 2–7).

Two guidelines did not have WHO crude suicide rates: Scotland^33^ and the Cook Islands.^52^ Four separate guidelines were reported on the aboriginal population, whereas New Zealand included specific recommendations for the Maori people within the general guideline.^44^ Only three guidelines focused family-based recommendations solely on children and adolescents: the Canadian guideline specifically for Aboriginal youth,^20^ Denmark^26^ and Croatia.^60^ The El Salvadorian^66^ guideline focused on both youth and pregnant women. Both Italian guidelines had prison-related target populations.^56,67^

### Family-based recommendations

**Fig. 2:**
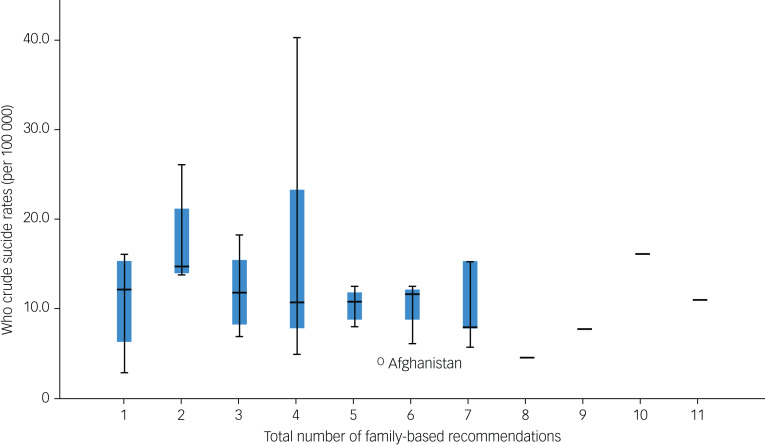
Box plot for total number of family-based recommendations by World Health Organization (WHO) crude suicide rates. The blue shaded areas represent the 2nd quartile and 3rd quartile.

The total number of family-based recommendations varied from 1 to 11 in the guidelines (Fig. 2). Countries with guidelines that have two recommendations have a median crude suicide rate (between 15 and 20 per 100 000 people) that is higher than countries with both more and, curiously, fewer recommendations. Very few countries have a total of 9 or more recommendations (Fig. 2). The greatest spread of WHO suicide rates is seen in countries with four recommendations.

The highest number of recommendations is seen in New Zealand^44^ and the USA,^57^ with a total of 11 and 10 recommendations respectively. The lowest number of recommendations is seen in the separate USA guideline specific to the American Indian/Alaskan Native population,^21^ Bulgarian^49^ and Panama^23^ guidelines, with one recommendation stated.

A listed of all family-based recommendations can be found in supplementary Tables 2–7.

### Categories of family-based recommendations

#### Prevention recommendations

**Fig. 3:**
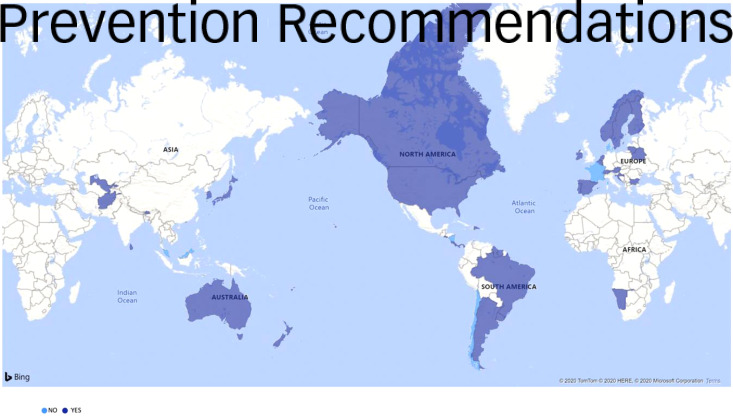
Prevention-focused family-based recommendations by country.

In total, 47 guidelines included any kind of family-based prevention recommendation. Specifically, 33 guidelines included education, awareness and psychoeducation-based prevention recommendations for families, 10 included prevention recommendations centred on building family-based resilience, 18 included prevention recommendations involving self-help groups and counselling, 30 guidelines mentioned other kinds of family-based prevention recommendations, and 5 guidelines did not report any prevention recommendations (Malaysia,^68^ Denmark,^26^ France,^50^ Chile^61^ and Nicaragua^69^) (Fig. 3).

#### Intervention recommendations

**Fig. 4:**
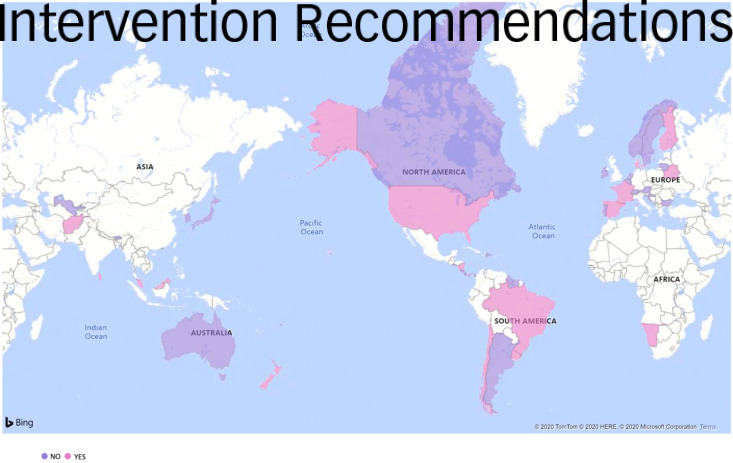
Intervention-focused family-based recommendations by country.

A total of 17 guidelines included any kind of family-based intervention recommendation: 13 of these included acute intervention recommendations and 6 included other intervention recommendations for immediate action. A total of 35 guidelines did not include any intervention recommendations (Fig. 4).

#### Postvention recommendations

**Fig. 5:**
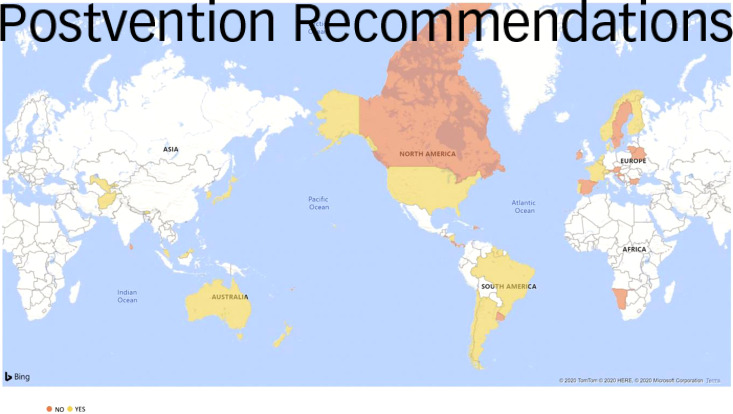
Postvention-focused family-based recommendations by country.

A total of 29 guidelines included postvention recommendations, all of which identified recommendations for families bereaved by suicide. Twenty-three countries did not include any postvention recommendations (Fig. 5).

**Fig. 6:**
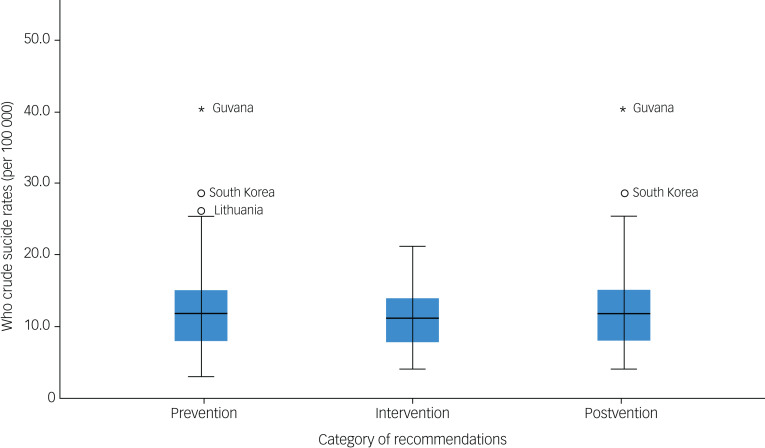
Box plot for categories of family-based recommendations by World Health Organization (WHO) crude suicide rates. The blue shaded areas represent the 2nd quartile and 3rd quartile.

Countries with prevention, intervention and postvention recommendations have similar WHO crude suicide rate medians. South Korea,^59^ Guyana^14^ and Lithuania^39^ all have prevention recommendations but also have high WHO crude suicide rates that resulted in them being identified as outliers. Guyana^14^ and South Korea^59^ have postvention recommendations but owing to their high WHO crude suicide rates, they were identified as outliers in the spread of rates in the postvention category (Fig. 6).

**Fig. 7:**
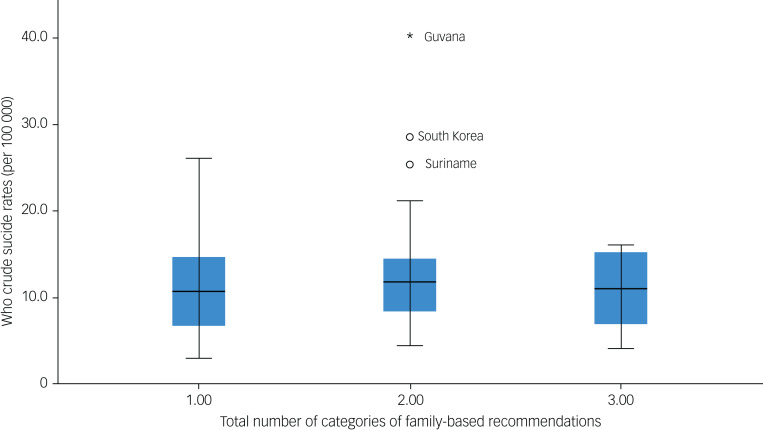
Box plot for total number of categories of family-based recommendations by World Health Organization (WHO) crude suicide rates. The blue shaded areas represent the 2nd quartile and 3rd quartile.

Countries with any two categories of family-based recommendations had a large number of outliers. Specifically, Guyana,^14^ South Korea^59^ and Suriname^46^ were identified as outliers for countries with any two categories of family-based recommendations. Countries with only one category of family-based recommendations had a large spread of crude suicide rates, nearing 30 per 100 000 people, in comparison with countries that had either any two or all three categories of family-based recommendations (Fig. 7).

### International guidelines

Two international guidelines were identified, one of which was the Euregenas (European Regions Enforcing Actions Against Suicide) guideline titled *General Guidelines on Suicide Prevention*, which was published and funded by the European Union from 2008 to 2013.^70^ Its rationale for the inclusion of family-based recommendations states that those bereaved by suicide are greatly affected. It establishes the importance of mental health promotion that provides family support.^70^ The second international guideline that was reported was published by the Pan American Health Organization and WHO in 2016.^71^ Family based-stigma and family history of suicide were presented as risk factors. These guidelines presented suicide data from America, Chile, the Dominican Republic, Mexico, Cuba, Nicaragua and Puerto Rico.^71^

### WHO crude suicide rates

The lowest crude rates of suicide were seen in Panama (2.9 per 100 000) and Afghanistan (4.1 per 100 000),^16^ but the WHO reports state this may be due to the underreporting of suicides. The highest crude rates were seen in Guyana (40.3 per 100 000) and South Korea (28.6 per 100 000).^16^ From the European region, Italy, Northern Ireland, Ireland, Spain, England, Bulgaria, The Netherlands, Denmark, Luxembourg, Portugal and Norway all have suicide rates below the regional average of 12.8 per 100 000.^16^ In the Americas, Panama, Brazil, Chile, Costa Rica, the Dominican Republic, Nicaragua and El Salvador have rates lower than the regional average of 9.6 per 100 000.^16^ Bhutan, Afghanistan, Malaysia and Uzbekistan have suicide rates lower than the Southeast Asia regional average of 10.1 per 100 000. Australia, New Zealand and Fiji have rates higher than the 8.2 per 100 000 regional average.

### Unpaired *t*-tests

All *t*-tests comparing the mean WHO crude suicide rates between countries that included and did not include family-based prevention, intervention and postvention recommendations yielded no significant results (*t* = 1.259, 0.712 and 0.423 respectively; *P* = 0.214, 0.480 and 0.674 respectively). A *t*-test was conducted comparing the crude suicide rates for countries with and without a family-based prevention recommendation, but it is important to note that only five countries did not include a prevention recommendation. Details regarding the independent *t*-tests can be found in Table 2.

**Table 2:** Unpaired *t*-tests comparing World Health Organization crude suicide rates between guidelines that did and did not include family-based prevention, intervention and postvention recommendations

Outcome	Mean (s.d.) per 100 000		*t* (d.f.)^a^	*P*	Mean difference (s.e. difference)	95% CI of the difference
**Family-based prevention recommendations**						
	Included (*n* = 45)	Did not include (*n* = 5)				
Crude suicide rates^b^	12.81 (7.11)	8.72 (3.79)	1.259 (48)	0.214	4.09 (3.25)	−2.44 to 10.62
**Family-based intervention recommendations**						
	Included (*n* = 16)	Did not include (*n* = 34)				
Crude suicide rates^b^	11.38 (5.29)	12.88 (7.61)	0.712 (48)	0.480	−1.50 (2.11)	−5.75 to 2.27
**Family-based postvention recommendations**						
	Included (*n* = 28)	Did not include (*n* = 22)				
Crude suicide rates^b^	12.77 (7.81)	11.93 (5.77)	0.423 (48)	0.674	0.843 (1.99)	−3.16 to 4.85

d.f., degrees of freedom.

a. Absolute values reported.

b. Equal variances assumed.

### One-way ANOVA

The one-way ANOVA conducted to compare the mean crude suicide rates between countries with guidelines that included one, two or three categories of family-based recommendations was found to be insignificant (Table 3). Thus, there was no statistically significant difference found between the mean crude suicide rates in the guidelines with only one, any two or all three categories of family-based recommendations (*F* = 0.751; between-group d.f. = 2, within-group d.f. = 47; *P* = 0.478).

**Table 3:** One-way ANOVA comparing the mean World Health Organization (WHO) crude suicide rates for only one, any two and all three categories of family-based recommendations

Number of categories of family-based recommendations (number of guidelines)	WHO crude suicide rate per 100 000, mean (s.d.)	95% CI of mean	*F* (between-group d.f., within-group d.f.)	*P*
Only one category (16)	11.09 (5.71)	8.04–14.13		
Any two categories (25)	13.42 (7.76)	10.48–16.38		
All three categories (5)	10.68 (5.20)	4.22–17.14		
			0.751 (2, 47)	0.478

d.f., degrees of freedom.

### Risk of bias within and across studies

Argentina,^51^ Belarus,^47^ the Cook Islands,^52^ Fiji^42^ and Panama^23^ all presented with the second lowest overall guideline domain score of about 33%, indicating poor quality. Sri Lanka^28^ presented with the lowest overall guideline score of about 16.7%, with the rigour of development domain scoring 6.25%, indicating poor quality. The highest overall guideline scores were seen for Australia,^30^ Belgium,^48^ Bhutan^72^ and Portugal,^73^ with scores of 75%. The lowest scope and purpose score was seen for the Cook Islands, with a score of 39%. The clarity of presentation domain had scores reaching as high as 61.1%, whereas the lowest scores were around 22.2% and were seen for Argentina^51^ and Sri Lanka.^28^ The applicability domain had scores varying from 16.7 to 62.5%, the highest of which was seen in the Scottish guideline.^33^ The domain of editorial independence had the lowest score (of 16.7%) for Sri Lanka^28^ and the highest score (of 58.3%) for the Dominican Republic.^55^ A complete summary of the risk of bias can be found in supplementary Table 8.

## Discussion

All identified guidelines included family-based recommendations. When these recommendations were categorised and assessed quantitatively, there was no significant difference in mean crude suicide rates between countries that did and did not have family-based prevention, intervention and postvention recommendations. There was also no significant difference found between the mean crude suicide rates of countries with guidelines that have only one, any two or all three categories of family-based recommendations. However, there were only five guidelines included in one subgroup in the ANOVA (guidelines that included all three categories of recommendations), which may limit the statistical accuracy of the output and provides reason to explore the descriptive differences between these groups. Specifically, countries with all three family-based prevention, intervention and postvention recommendations had a smaller spread of crude suicide rates and lower mean crude suicide rate when compared with guidelines that included only one or any two categories of family-based recommendations. This may be indicative of the importance of including all three categories of family-based recommendations in guidelines to effectively mitigate high crude suicide rates. This is supported by a WHO report on suicide prevention, which outlines the importance of a comprehensive suicide strategy approach, one that consists of multi-level prevention, intervention and postvention recommendations.^74^

Although all guidelines identified included family-based recommendations, some included a limited number of recommendations and were often lacking thorough rationale and implementation measures. More specifically, the European countries Austria,^53^ Belarus,^47^ Bulgaria,^31^ France,^50^ Lithuania^39^ and Sweden^41^ listed only one or two family-based recommendations. All of the these countries, excluding Bulgaria,^31^ also happen to have crude suicide rates close to or above the European regional rate of 12.8 per 100 000 people (Austria 14.6 per 100 000, Belarus 21.2 per 100 000, France 13.8 per 100 000, Lithuania 26.1 per 100 000, Sweden 14.7 per 100 000).^16^ Thus, in many of the countries with a lower number of reported family-based recommendations, there is reason to suggest that this lower number is associated with higher-than-average suicide rates. Bulgaria (9.7 per 100 000) was the only one of these countries that included an action plan, and thus it may have presented as an exception to the association owing to the country's inclusion of specific implementation and effectiveness measures and indicators as outlined in the action plan.

In contrast, the USA^48^ and New Zealand^44^ were the countries with the highest number of included family-based recommendations, with 10 recommendations in the US guidelines and 11 in those from New Zealand. Despite the high number of recommendations, both countries present crude suicide rates that are above their regional averages: the USA has a rate of 16.1 per 100 000 and a regional average of 9.6 per 100 000, and New Zealand has a rate of 11.0 per 100 000 and a regional average of 10.1 per 100 000.^16^ This finding brings forth an important consideration on the quality of the included interventions. More specifically, the rationale included in each guideline ranged from two to three points about family as a protective and risk factor for suicide, and ultimately the included recommendations were not presented with in-depth discussions of supporting evidence. Thus, although these countries included a large number of family-based recommendations, the lack of focus on the supporting evidence for these recommendations may have compromised the quality of the proposed recommendations. This is supported by a WHO global report looking at national suicide recommendations which mentioned that for countries like the USA and New Zealand, which have fairly comprehensive national responses, the focus should be on evaluation and improvement of listed recommendations.^74^ This further emphasises the importance of quality assessment of the family-based recommendations included in national suicide guidelines, to ensure that the recommendations improve in effectiveness and sustainability over time.

This inconsistency in the evidence included in guidelines and the associated recommendations that are presented is also evident in the lack of recommendations based on familial risk factors. A total of 32 guidelines listed familial risk factors for suicide, whereas a limited number included recommendations that directly addressed risk factors such as family history of suicide. For example, the Australian guideline looking at the Torres Strait Islander population^62^ specifically mentioned the disproportionately large number of deaths by suicide where there was a history of childhood separation and abandonment. However, there are no Australian recommendations included that address the country-specific rationale provided for the inclusion of family-based recommendations. Furthermore, ten guidelines did not include any rationale for their inclusion of family-based recommendations. Thus, there is a level of inconsistency between the evidence used to support family-based recommendations in these guidelines and the actual recommendations that are implemented. It may be beneficial for countries to focus not only on presented rationale for the inclusion of family in recommendations but also to tailor specific recommendations to the evidence being presented in order to increase effectiveness of recommendations. One specific example of this phenomenon is seen with Guyana,^14^ which listed high percentages of family discord, relationship problems, domestic violence and interpersonal conflict, whereas its recommendations were mostly focused on those bereaved by suicide. Thus, the lack of inclusion of recommendations that target the listed familial risk factors may ultimately be contributing to the high rate of suicide seen in the Guyanese population (40.3 per 100 000),^16^ providing support for the evidence-based tailoring of recommendations included in national suicide guidelines.

It is also important to mention that Namibia^84^ was the only African country identified with a national suicide guideline, a situation confirmed by a report on national suicide guidelines published by the WHO.^74^ Namibia included five family-based recommendations and four points in its rationale for including these recommendations. Despite having a national suicide guideline, and also including family-based recommendations, Namibia has a suicide rate of 9.7 per 100 000, which is higher than the African average of 6.9 per 100 000.^74^ Although the Namibian suicide rate has been fluctuating over the years, it has been steadily declining since 2017, whereas for the years preceding the publication of the 2012 national guideline (e.g. 2005 and 2010), the crude suicide rate remained the same.^85,86^ Thus, the inclusion of family-based recommendations and the establishment and progression of a national suicide guideline may contribute to the decline in suicide rate seen in Namibia.

### Limitations

Despite efforts to minimise them, this review has a number of limitations. It is important to note that how guidelines are implemented will have a strong impact on suicide rates: although guidelines may present high-quality interventions, the level of implementation and efficacy of the interventions will affect outcomes. Future directions should involve an assessment of Delphi studies and other reports that analyse the success of the family-based recommendations included in the guidelines. Furthermore, suicide is a highly complex outcome that is influenced by numerous other factors in each country, such as biological, economic and social factors. Thus, the rates of suicide may not be reflective of the interventions included in a country's guideline and will be influenced by these factors as well. It is also important to mention that the search strategies were carried out in English, thus limiting the number of guidelines obtained from non-English speaking countries. The inclusion of grey literature and manual search strategies were used to mitigate this limitation. Furthermore, there were national guidelines identified that referred to specific groups of people that may have varying rates of suicide compared with the national averages obtained from the WHO. The inclusion of these guidelines is justified by their representation of national suicide-specific recommendations, despite the limitations present in obtaining group-specific national suicide rates. It was important to ensure that these guidelines were included and recognised in this review as they fall under the inclusion criteria and highlight populations that various countries consider at risk. Additionally, in this review we used the WHO crude suicide rates from the year 2019 for ease of comparison between national guidelines. However, the impact of the guidelines on suicide rates may vary, as they were published in different years. Also, the rates obtained from the WHO may not be comprehensive, but justification of their use is in the fact that the WHO maintains one of the few global databases that can be used in a review such as this one, that spans multiple geographical locations. Lastly, a limitation in this review is that the mention of family could have led to the introduction of heterogeneity due to cultural differences between countries and may not be directly related to the geographical location.

### Recommendations

Future research should include an investigation of empirical literature evaluating the guidelines with family-based recommendations, possibly leading to the completion of further quantitative analyses to investigate the associations between family-based recommendations and suicide rates in greater detail. Furthermore, the unequal numbers of countries with guidelines per region is a reason for the development and implementation of national suicide guidelines in more countries, especially in Africa. Overall, based on our findings, countries should be working towards the development and implementation of comprehensive national suicide guidelines that include family-based recommendations focused on prevention, intervention and postvention, with the corresponding implementation plans to potentially mitigate suicide. Countries should also acknowledge family as a protective and risk factor in the rationale for the recommendations they include and should focus their efforts on the inclusion of country-specific and evidence-based recommendations.

## Data Availability

The data used in this review has not been posted on a public platform. The data that support the findings of this study are available from the corresponding author, Z.S., upon reasonable request.

## Supplementary material

For supplementary material accompanying this paper visit http://doi.org/10.1192/bjo.2022.15. click here to view supplementary material
