# Supplementary material for: National suicide management guidelines recommending family-based prevention, intervention and postvention and their association with suicide mortality rates: systematic review
Source: BJPsych Open. 2022 Feb 24;8(2):e54. doi: 10.1192/bjo.2022.15 (PMC8935913; doi:10.1192/bjo.2022.15)
Supplement: Supplementary file 1 [file S2056472422000151sup001.docx]

Supplemental File

Supplemental table 1.0: Summary of the categories of recommendations, evidence for recommendations, and implementation and effectiveness measures by country

| Country | Family-Based Prevention | education-based prevention | resilience-based prevention | counselling-based prevention | other prevention | Family-Based Intervention | acute intervention | other intervention | Family-Based Postvention | bereavement postvention | other postvention | Total Number of Family Recommendations | Evidence for family as a risk factor | Evidence for family as a protective factor | other evidence for family-based recommendations | Total Number of Evidence for family-based recommendations | Measures of implementation | Measures of effectiveness | WHO Crude Suicide Rates (per 100 000) |
| --- | --- | --- | --- | --- | --- | --- | --- | --- | --- | --- | --- | --- | --- | --- | --- | --- | --- | --- | --- |
| Afghanistan | 3 | 1 | 0 | 1 | 1 | 1 | 0 | 1 | 1 | 1 | 0 | 5 | 1 | 0 | 1 | 2 | 2 | 0 | 4.1 |
| Bhutan | 7 | 2 | 1 | 2 | 2 | 0 | 0 | 0 | 1 | 1 | 0 | 8 | 4 | 3 | 0 | 7 | 1 | 2 | 4.6 |
| Japan | 3 | 1 | 0 | 1 | 1 | 0 | 0 | 0 | 4 | 3 | 1 | 7 | 1 | 0 | 0 | 1 | 2 | 2 | 15.3 |
| Malaysia | 0 | 0 | 0 | 0 | 0 | 5 | 3 | 2 | 2 | 2 | 0 | 7 | 1 | 1 | 0 | 2 | 1 | 3 | 5.7 |
| South Korea | 3 | 1 | 0 | 1 | 1 | 0 | 0 | 0 | 1 | 1 | 0 | 4 | 3 | 0 | 1 | 4 | 2 | 2 | 28.6 |
| Sri Lanka | 1 | 0 | 0 | 0 | 1 | 1 | 0 | 1 | 0 | 0 | 0 | 2 | 0 | 0 | 1 | 1 | 2 | 0 | 14 |
| Uzbekistan | 4 | 2 | 1 | 0 | 1 | 0 | 0 | 0 | 1 | 1 | 0 | 5 | 2 | 1 | 0 | 3 | 1 | 1 | 8 |
| Austria | 1 | 0 | 0 | 0 | 1 | 0 | 0 | 0 | 0 | 0 | 0 | 1 | 0 | 0 | 1 | 1 | 1 | 1 | 14.6 |
| Belarus | 1 | 0 | 0 | 1 | 0 | 1 | 0 | 1 | 0 | 0 | 0 | 2 | 0 | 0 | 0 | 0 | 2 | 1 | 21.2 |
| Belgium | 2 | 0 | 1 | 0 | 1 | 0 | 0 | 0 | 1 | 1 | 0 | 3 | 0 | 0 | 0 | 0 | 1 | 1 | 18.3 |
| Bulgaria | 1 | 1 | 0 | 0 | 0 | 0 | 0 | 0 | 0 | 0 | 0 | 1 | 0 | 0 | 0 | 0 | 1 | 1 | 9.7 |
| Croatia | 3 | 1 | 0 | 1 | 1 | 0 | 0 | 0 | 0 | 0 | 0 | 3 | 1 | 0 | 1 | 2 | 3 | 1 | 16.4 |
| Denmark | 0 | 0 | 0 | 0 | 0 | 3 | 1 | 2 | 1 | 1 | 0 | 4 | 2 | 1 | 0 | 3 | 1 | 0 | 10.7 |
| England | 2 | 0 | 0 | 1 | 1 | 0 | 0 | 0 | 5 | 5 | 0 | 7 | 1 | 0 | 1 | 2 | 1 | 2 | 7.9 |
| Finland | 5 | 1 | 0 | 0 | 4 | 1 | 1 | 0 | 1 | 1 | 0 | 7 | 1 | 2 | 1 | 4 | 1 | 1 | 15.3 |
| France | 0 | 0 | 0 | 0 | 0 | 1 | 1 | 0 | 1 | 1 | 0 | 2 | 0 | 0 | 0 | 0 | 1 | 2 | 13.8 |
| Ireland | 3 | 3 | 0 | 0 | 0 | 0 | 0 | 0 | 2 | 2 | 0 | 5 | 2 | 1 | 0 | 3 | 1 | 1 | 9.6 |
| Italy (adult penitentiary) | 4 | 0 | 0 | 0 | 4 | 0 | 0 | 0 | 0 | 0 | 0 | 4 | 0 | 1 | 0 | 1 | 1 | 1 | 6.7 |
| Italy (youth penitentiary) | 4 | 0 | 0 | 0 | 4 | 0 | 0 | 0 | 0 | 0 | 0 | 4 | 2 | 1 | 1 | 4 | 1 | 1 | 6.7 |
| Lithuania | 2 | 0 | 0 | 0 | 2 | 0 | 0 | 0 | 0 | 0 | 0 | 2 | 1 | 0 | 1 | 2 | 1 | 1 | 26.1 |
| Luxembourg | 3 | 1 | 2 | 0 | 0 | 1 | 1 | 0 | 0 | 0 | 0 | 4 | 1 | 1 | 0 | 2 | 1 | 2 | 11.3 |
| Netherlands | 3 | 2 | 0 | 1 | 0 | 0 | 0 | 0 | 2 | 2 | 0 | 5 | 3 | 2 | 0 | 5 | 0 | 2 | 11.8 |
| Northern Ireland | 2 | 2 | 0 | 0 | 0 | 0 | 0 | 0 | 5 | 5 | 0 | 7 | 2 | 1 | 0 | 3 | 2 | 2 | 7.9 |
| Norway | 4 | 2 | 0 | 2 | 0 | 0 | 0 | 0 | 2 | 1 | 1 | 6 | 1 | 1 | 2 | 4 | 1 | 0 | 11.8 |
| Portugal | 5 | 0 | 1 | 1 | 3 | 0 | 0 | 0 | 1 | 1 | 0 | 6 | 2 | 1 | 6 | 9 | 2 | 1 | 11.5 |
| Scotland | 1 | 1 | 0 | 0 | 0 | 1 | 0 | 1 | 1 | 1 | 0 | 3 | 0 | 0 | 0 | 0 | 1 | 1 |  |
| Spain | 6 | 2 | 0 | 0 | 4 | 3 | 3 | 0 | 0 | 0 | 0 | 9 | 2 | 0 | 0 | 2 | 1 | 0 | 7.7 |
| Sweden | 2 | 1 | 0 | 0 | 1 | 0 | 0 | 0 | 0 | 0 | 0 | 2 | 0 | 0 | 0 | 0 | 1 | 2 | 14.7 |
| Switzerland | 2 | 1 | 0 | 1 | 0 | 0 | 0 | 0 | 1 | 1 | 0 | 3 | 1 | 0 | 1 | 2 | 1 | 3 | 14.5 |
| Namibia | 3 | 2 | 0 | 1 | 0 | 2 | 2 | 0 | 0 | 0 | 0 | 5 | 1 | 1 | 2 | 4 | 1 | 1 | 9.7 |
| Australia | 5 | 1 | 1 | 1 | 2 | 0 | 0 | 0 | 1 | 1 | 0 | 6 | 1 | 1 | 0 | 2 | 2 | 5 | 12.5 |
| Australia (aboriginal) | 3 | 1 | 1 | 0 | 1 | 0 | 0 | 0 | 2 | 2 | 0 | 5 | 2 | 0 | 0 | 2 | 1 | 1 | 12.5 |
| New Zealand | 8 | 2 | 2 | 0 | 4 | 1 | 1 | 0 | 2 | 2 | 0 | 11 | 0 | 1 | 1 | 2 | 1 | 2 | 11 |
| Cook’s Island | 4 | 0 | 0 | 1 | 3 | 0 | 0 | 0 | 0 | 0 | 0 | 4 | 0 | 0 | 1 | 1 | 1 | 3 |  |
| Fiji | 4 | 2 | 0 | 0 | 2 | 0 | 0 | 0 | 0 | 0 | 0 | 4 | 0 | 0 | 0 | 0 | 1 | 2 | 9 |
| Argentina | 2 | 0 | 0 | 0 | 2 | 0 | 0 | 0 | 1 | 1 | 0 | 3 | 0 | 0 | 0 | 0 | 1 | 1 | 8.4 |
| Brazil | 1 | 1 | 0 | 0 | 0 | 1 | 1 | 0 | 1 | 1 | 0 | 3 | 0 | 0 | 0 | 0 | 2 | 2 | 6.9 |
| Chile | 0 | 0 | 0 | 0 | 0 | 3 | 3 | 0 | 1 | 1 | 0 | 4 | 4 | 0 | 3 | 7 | 2 | 1 | 9 |
| Guyana | 2 | 1 | 0 | 1 | 0 | 0 | 0 | 0 | 2 | 2 | 0 | 4 | 1 | 0 | 4 | 5 | 4 | 2 | 40.3 |
| Suriname | 1 | 1 | 0 | 0 | 0 | 0 | 0 | 0 | 3 | 3 | 0 | 4 | 4 | 2 | 0 | 6 | 2 | 3 | 25.4 |
| Uruguay | 3 | 1 | 0 | 0 | 2 | 1 | 1 | 0 | 0 | 0 | 0 | 4 | 0 | 1 | 0 | 1 | 1 | 2 | 21.2 |
| Canada | 5 | 4 | 1 | 0 | 0 | 0 | 0 | 0 | 0 | 0 | 0 | 5 | 0 | 0 | 0 | 0 | 1 | 1 | 11.8 |
| Canada (National Inuit Strategy) | 3 | 1 | 1 | 0 | 1 | 0 | 0 | 0 | 0 | 0 | 0 | 3 | 2 | 0 | 3 | 5 | 1 | 1 | 11.8 |
| Canada (armed forces) | 4 | 1 | 0 | 1 | 2 | 0 | 0 | 0 | 1 | 1 | 0 | 5 | 2 | 0 | 0 | 2 | 0 | 1 | 11.8 |
| Canada (Aboriginal youth) | 3 | 1 | 0 | 0 | 2 | 0 | 0 | 0 | 0 | 0 | 0 | 3 | 2 | 0 | 0 | 2 | 1 | 0 | 11.8 |
| Costa Rica | 3 | 0 | 0 | 3 | 0 | 0 | 0 | 0 | 0 | 0 | 0 | 3 | 1 | 0 | 0 | 1 | 2 | 1 | 8.1 |
| Dominican Republic | 4 | 2 | 0 | 0 | 2 | 0 | 0 | 0 | 0 | 0 | 0 | 4 | 0 | 1 | 0 | 1 | 1 | 4 | 4.9 |
| El Salvador | 6 | 2 | 0 | 4 | 0 | 0 | 0 | 0 | 0 | 0 | 0 | 6 | 2 | 0 | 0 | 2 | 1 | 1 | 6.1 |
| Nicaragua | 0 | 0 | 0 | 0 | 0 | 7 | 7 | 0 | 1 | 1 | 0 | 8 | 2 | 0 | 1 | 3 | 1 | 1 | 4.4 |
| Panama | 1 | 0 | 0 | 0 | 1 | 0 | 0 | 0 | 0 | 0 | 0 | 1 | 0 | 0 | 1 | 1 | 1 | 0 | 2.9 |
| United States of America | 4 | 4 | 0 | 0 | 0 | 2 | 2 | 0 | 4 | 1 | 3 | 10 | 0 | 2 | 1 | 3 | 2 | 5 | 16.1 |
| United States of America (American Indian) | 1 | 0 | 0 | 0 | 1 | 0 | 0 | 0 | 0 | 0 | 0 | 1 | 0 | 0 | 1 | 1 | 1 | 0 | 16.1 |

| Supplemental table 2.0: Guidelines from Asia | |
| --- | --- |
| Afghanistan^25^ | |
| Year | 2018-2022 |
| Title | National Suicide Prevention Strategy |
| Population of Interest | General |
| Family Strategy(ies) | Yes |
| Prevention | |
| Suicide-related awareness, education, psychoeducation for families | - Enhance awareness of availability of hazardous means for suicide, and promote alertness of families and communities toward suicide by spreading information about the possible emergency responses |
| Increase familial resilience | Not reported |
| Family-based psychosocial counselling | - Psychosocial counselling and legal counselling for women who have been victims of familial violence: 16 shelters offer these services and they are located in provinces where victims have been protected |
| Other preventions | - Involve family/parents of students to lower rates of student dropout |
| Intervention | |
| Acute family-related intervention strategies | Not reported |
| Other interventions | - Strengthening non-pharmacological treatment for drug users, such as motivational counselling and establishment of family support |
| Postvention | |
| Support for families bereaved by suicide | - Establish functioning of psychiatric care to support those bereaved by suicide |
| Other postventions | Not reported |
| Guideline’s rationale for including family-based intervention(s) in recommendations | |
| Family as a risk factor | - Most risk factors of suicide for women are forced marriages, early child marriages, multiple marriages, lack of knowledge of rights, psychological impact of 25 years of war, and family violence |
| Family as a protective factor | Not reported |
| Other | - The variations in the patterns of death by suicide over time indicate the influence of cultural and religious factors, strong familial ties, and general social structure |
| Guideline’s measures of implementation and effectiveness of family-based intervention(s) | |
| Measures of Implementation | - Technical agencies and other stakeholders work towards implementing the suicide prevention strategy through intersectional collaboration   - These organizations are, High court, general attorney office, WHO, gender section of United Nation Fund for Family and Population (UNFPA), European Union, civil society and other relevant government - Collaboration is very important because suicide is a criminal act by SHAREA law, civil and criminal law so more advocacy is needed to decriminalize it |
| Measures of Effectiveness | Not reported |
| Inclusion of action plan or progress reported | Not reported |
| Rate of suicide from WHO | 4.7 per 100 000 |
| Bhutan^75^ | |
| Year | July 2015 – June 2018 |
| Title | Suicide prevention in Bhutan – A 3-year action plan |
| Population of Interest | General |
| Family Strategy(ies) | Yes |
| Prevention | |
| Suicide-related awareness, education, psychoeducation for families | - Parents encouraged to take ownership of children’s education in order to understand their needs and address psychopathology the children may be experiencing - Cluster community program is a program for armed forces families to help learn way to support their children |
| Increase familial resilience | - Community resilience and support programs are important to build broader suicide prevention services   - Village health workers (VHWs) stimulate community engagement and improve family planning and immunization services |
| Family-based psychosocial counselling | - Social and family therapy and counseling services are not available in the country but the Chithuen Phendey Association (CPA) is working towards administering a counselling program for families and addicts - Counsellor competency will be updated to address families |
| Other preventions | - National Commission of Women and Children (NCWC) instituted one stop crisis center for women and children who are victims of domestic violence, potentially directly reaching individuals in crisis and at risk of suicide - Health service providers are mandated to provide 24 hours of service to the victims of domestic violence |
| Intervention | |
| Acute family-related intervention strategies | Not reported |
| Other interventions | Not reported |
| Postvention | |
| Support for families bereaved by suicide | - Encourage culturally appropriate community bereavement support for families and others impacted by suicide through the development of a postvention protocol for health workers, police, school counselors and district response teams |
| Other postventions | Not reported |
| Guideline’s rationale for including family-based intervention(s) in recommendations | |
| Family as a risk factor | - Family history of suicide, poor family and personal relationships as risk factors - 2496 cases of student counseling in offered in 52 schools in 2014 where 301 were for family issues - Harmful use of alcohol is linked to suicide in Bhutan and most of these individuals are found to have poor family support - Domestic violence, and children who have experienced child abuse often at greater risk of death by suicide |
| Family as a protective factor | - Deep family bonds, religious beliefs and positive cultural values in the communities are social capital to boost suicide prevention services in the communities - Strong family bonding, shared family values as a social fabric of Bhutanese society - Increasing resilience, resourcefulness, respect, and interconnectedness for individuals, families, and communities is a positive factor for suicide prevention |
| Other | Not reported |
| Guideline’s measures of implementation and effectiveness of family-based intervention(s) | |
| Measures of Implementation | - 29 various organizations graded suicide prevention strategies on criteria such as feasibility; anything scoring a 60% or higher was included in the action plan as deliverable and actionable prevention strategies |
| Measures of Effectiveness | - Goal is to have 50% of suicide survivors receive crisis counseling and suicide risk assessment by a trained professional (health worker, school counselor or peer counselors) - And 80% of the bereaved families to get support from the neighbors and communities |
| Inclusion of action plan or progress reported | - Progress of plan assessed yearly, with full plan evaluation occurring in 2018 |
| Rate of suicide from WHO | 11.4 per 100 000 |
| Japan^66^ | |
| Year | 2017 |
| Title | The General Principles of Suicide Prevention Policy: Realizing a Society in Which No One Is Driven to Take Their Own Life |
| Population of Interest | General |
| Family Strategy(ies) | yes |
| Prevention | |
| Suicide-related awareness, education, psychoeducation for families | - Strengthen support for nursing mothers to prevent post partum depression; visit all babies up to four months old to provide extra information on parenting support |
| Increase familial resilience | Not reported |
| Family-based psychosocial counselling | - Hikikomori centres provide counselling services for people in acute social withdrawal |
| Other preventions | - Ministry of Health, Labour and Welfare to provide assistance to caregivers of people at risk |
| Intervention | |
| Acute family-related intervention strategies | Not reported |
| Other interventions | Not reported |
| Postvention | |
| Support for families bereaved by suicide | - Minimize the impact on family members, co-workers and others who have been left behind and prevent new suicides from happening by promoting self-help groups, better personnel dealing with families, and supporting bereaved children - Improve quality of personnel at public agencies who deal with bereaved family members and others - Promote the dissemination of knowledge about how to deal appropriately with bereaved family members |
| Other postventions | - Improve the counseling system for those who have survived a suicide attempt as provided by public health nurses at mental health and welfare centers and public health centers |
| Guideline’s rationale for including family-based intervention(s) in recommendations | |
| Family as a risk factor | - Family problems and issues as a risk factor for suicide in general population, survivors of domestic violence and people with alcohol use disorder |
| Family as a protective factor | Not reported |
| Other | Not reported |
| Guideline’s measures of implementation and effectiveness of family-based intervention(s) | |
| Measures of Implementation | - Collaboration and coordination is needed among policy measures, people and organizations in a variety of fields |
| Measures of Effectiveness | - Promoting research and studies that will contribute to the promotion of comprehensive suicide countermeasures - Conduct studies to obtain a multifaceted understanding of the reasons for, background to and the process that leads up to suicide |
| Inclusion of action plan or progress reported | Basic Law on Suicide Countermeasures for Japan is also published and it mandates suicide prevention and support for relatives and others when advocating for suicide prevention^38^ |
| Rate of suicide from WHO | 18.5 per 100 000 |
| Malaysia^71^ | |
| Year | 2013 |
| Title | Guideline on suicide prevention and management |
| Population of Interest | General |
| Family Strategy(ies) | Yes |
| Prevention | |
| Suicide-related awareness, education, psychoeducation for families | Not reported |
| Increase familial resilience | Not reported |
| Family-based psychosocial counselling | Not reported |
| Other preventions | Not reported |
| Intervention | |
| Acute family-related intervention strategies | - If there is a low risk of suicide, then allow family member to wait while monitoring; medium risk then encourage family member to accompany in medical or surgical ward - Settling personal affairs, tying up loose ends or saying bye to family or friends; or asking for forgiveness should be seen as early detection points - When suicide attempt has occurred, healthcare profession should administer immediate communication with listed family (or friend) contact |
| Other interventions | - When an inpatient suicide occurs, continue conversations with family and when breaking bad news determine how much the family want to know before debriefing - Identify risk factors in screening processes (e.g. family history of suicide, family breakdown, custody issues) to assess level of risk |
| Postvention | |
| Support for families bereaved by suicide | - Offer staff and family members psychological services when someone has died by suicide - Community Mental Health Centres (Mentari) are walk-in centers with doctors and counselors for mental health issues and should offer support to friends and family of the victims |
| Other postventions | Not reported |
| Guideline’s rationale for including family-based intervention(s) in recommendations | |
| Family as a risk factor | - Family history of suicide, family breakdown, child custody issues are risk factors |
| Family as a protective factor | - Person’s support system (family, friends) has to be identified and put into action to reduce the risk |
| Other | Not reported |
| Guideline’s measures of implementation and effectiveness of family-based intervention(s) | |
| Measures of Implementation | - Organizations like Befrienders, Malaysian Mental Health Association; and Talian Nur under the Ministry of Women, Family and Community Development will implement bereavement support |
| Measures of Effectiveness | - Monitoring and assessing program include research such as:   - Epidemiological studies to identify high risk groups and changes in suicide behaviour overtime   - Studies assessing interaction between biological, psychological and social risk factors   - Studies assessing effectiveness of intervention and postvention services |
| Inclusion of action plan or progress reported | Not reported |
| Rate of suicide from WHO | 5.5 per 100 000 |
| South Korea (Republic of Korea) ^62^ | |
| Year | 2018 |
| Title | Suicide prevention action plan |
| Population of Interest | General |
| Family Strategy(ies) | Yes |
| Prevention | |
| Suicide-related awareness, education, psychoeducation for families | - Suicide Prevention Gatekeeper: Trained to quickly recognize and connect with experts when family and other factors trigger suicidal behaviour or ideation |
| Increase familial resilience | Not reported |
| Family-based psychosocial counselling | - Provide elderly with family counselling |
| Other preventions | - Promoting suicide prevention programs for job seekers and job centre support for unemployed families |
| Intervention | |
| Acute family-related intervention strategies | Not reported |
| Other interventions | Not reported |
| Postvention | |
| Support for families bereaved by suicide | - Strengthening support for families who are bereaved by suicide by having designated welfare centres and psychological counselling and self help groups that meet once a month |
| Other postventions | Not reported |
| Guideline’s rationale for including family-based intervention(s) in recommendations | |
| Family as a risk factor | - Suicide risk of suicidal families is 8.3 times that of the general population, and 41.7% experience depression - Depression, decreased motivation (75.0%), insomnia (69.4%), anxiety (65.3%), anger (63.9%) Mental symptoms, depression (41.7%), insomnia (37.5%), anxiety disorder (31.9%), adaptation disorder (23.6%), etc. are often associated with suicide risk - Low social cohesion and weakened family relations are pointed out as the main causes of high suicide rates. |
| Family as a protective factor | Not reported |
| Other | - Negative impact of pain on bereaved families is important to consider |
| Guideline’s measures of implementation and effectiveness of family-based intervention(s) | |
| Measures of Implementation | - Identify and put forth strategies that can be implemented in the short term to ensure feasibility and effectiveness - Establish Suicide Implementation Plan for local governments |
| Measures of Effectiveness | - Emphasize and spread the importance of research and development to evaluate program effectiveness - Evidence-based execution is emphasized through surveys |
| Inclusion of action plan or progress reported | - Act for the prevention of suicide and the creation of culture of respect for life includes a timeline to execute initiatives included in action plan ^37^   - Article 20 (Assistance to Persons who Attempted Suicide, etc.) The central government and each local government may provide psychological counseling and counseling treatment to persons who attempted suicide and to family members to help them minimize impact |
| Rate of suicide from WHO | 26.9 per 100 000 |
| Sri Lanka^31^ | |
| Year | 1997 |
| Title | National policy and action plan on prevention of suicide |
| Population of Interest | General |
| Family Strategy(ies) | Yes |
| Prevention | |
| Suicide-related awareness, education, psychoeducation for families | Not reported |
| Increase familial resilience | Not reported |
| Family-based psychosocial counselling | Not reported |
| Other preventions | To help families dealing with suicide, set-up social support centres at the community level and reach out to social workers for the community |
| Intervention | |
| Acute family-related intervention strategies | Not reported |
| Other interventions | Mechanisms set-up to intervene and help families with problems related to suicide by identifying the specific problems and contacting appropriate resources |
| Postvention | |
| Support for families bereaved by suicide | Not reported |
| Other postventions | Not reported |
| Guideline’s rationale for including family-based intervention(s) in recommendations | |
| Family as a risk factor | Not reported |
| Family as a protective factor | Not reported |
| Other | - Providing families who are at risk of suicide with community support should be done on the small scale and in an unpublicized manner   - This is done to minimize the belief that suicide is a logical way to solve one’s problems and to prevent families from ingesting poison solely for the reason to access community services |
| Guideline’s measures of implementation and effectiveness of family-based intervention(s) | |
| Measures of Implementation | - Training and sensitizing relevant people and agencies to collect and organize existing and new data - Assessment of both national and local measures carried out experimentally by trained academically oriented agencies |
| Measures of Effectiveness | Not reported |
| Inclusion of action plan or progress reported | Not reported |
| Rate of suicide from WHO | 14.6 per 100 000 |
| Uzbekistan^41^ | |
| Year | 2010-2020 |
| Title | Strategy for suicide prevention in the republic of Uzbekistan |
| Population of Interest | General |
| Family Strategy(ies) | Yes |
| Prevention | |
| Suicide-related awareness, education, psychoeducation for families | - Parliament adopted the "Program to improve the socio-psychological climate in the families of the republic"   - Promotion of a conflict-free family environment   - Increase the knowledge of the population to lower the number of suicidal attempts     - Staff of makhallas and educational institutions conduct lectures, and answer questions |
| Increase familial resilience | - Regulation and services work towards reducing the population's exposure to social, family and personal risk factors leading to risk of suicidal behavior by promoting mental health, well-being and resilience of families and individuals |
| Family-based psychosocial counselling | Not reported |
| Other preventions | - Means restriction: call on the vigilance of family and friends of individuals who have attempted suicide to restrict their access to suicide weapons. |
| Intervention | |
| Acute family-related intervention strategies | Not reported |
| Other interventions | Not reported |
| Postvention | |
| Support for families bereaved by suicide | - Healthcare professionals told to support families affected by suicide or attempted suicide |
| Other postventions | Not reported |
| Guideline’s rationale for including family-based intervention(s) in recommendations | |
| Family as a risk factor | - Poor mental health is a risk factor for suicidal behavior at any age, but factors related to family and trauma contribute even more to the etiology of youth suicide than adults - A substantial body of evidence shows that social, family and personality risk factors contribute to suicidal behavior and mental illness |
| Family as a protective factor | - Family support listed as an evidence based protective factor |
| Other | Not reported |
| Guideline’s measures of implementation and effectiveness of family-based intervention(s) | |
| Measures of Implementation | - Local implementation: The community-based approach to suicide aims to support and engage community; interdepartmental regional programs for the prevention of suicides have already been developed and are being implemented |
| Measures of Effectiveness | - Stakeholder performances will be monitored and changed based on the effectiveness of the activities as assessed by regular data collection and studies |
| Inclusion of action plan or progress reported | - In order to translate this strategy into concrete and controlled actions, the Ministry of Health will coordinate the development of republican and regional action plans, which will be periodically updated |
| Rate of suicide from WHO | Not reported |
| Supplemental table 3.0: Guidelines identified from Europe | |
| Austria^56^ | |
| Year | 2011 |
| Title | Suicide Prevention in Austria |
| Population of Interest | General |
| Family Strategy(ies) | Yes |
| Prevention | |
| Suicide-related awareness, education, psychoeducation for families | Not reported |
| Increase familial resilience | Not reported |
| Family-based psychosocial counselling | Not reported |
| Other preventions | - Public relations facilities help work on familial problems |
| Intervention | |
| Acute family-related intervention strategies | Not reported |
| Other interventions | Not reported |
| Postvention | |
| Support for families bereaved by suicide | Not reported |
| Other postventions | Not reported |
| Guideline’s rationale for including family-based intervention(s) in recommendations | |
| Family as a risk factor | Not reported |
| Family as a protective factor | Not reported |
| Other | - Traumatization of death by suicide for family members is significant |
| Guideline’s measures of implementation and effectiveness of family-based intervention(s) | |
| Measures of Implementation | - Collaboration between local programs and projects and crisis intervention facilities in Vienna to encourage training and development and increase national expertise |
| Measures of Effectiveness | Research division for mental health at the University for Medical Information and Technology (UMIT) work to assess policies and programs |
| Inclusion of action plan or progress reported | Not reported |
| Rate of suicide from WHO | 15.6 per 100 000 |
| Belarus^50^ | |
| Year | 2015-2019 |
| Title | A complex of measures for prevention of suicidal behavior of the population of the republic of Belarus  For 2015-2019 |
| Population of Interest | General |
| Family Strategy(ies) | Yes |
| Prevention | |
| Suicide-related awareness, education, psychoeducation for families | Not reported |
| Increase familial resilience | Not reported |
| Family-based psychosocial counselling | - Psychological assistance to citizens and their families through counselling |
| Other preventions | Not reported |
| Intervention | |
| Acute family-related intervention strategies | Not reported |
| Other interventions | - Interventions should address risk groups and support both individuals and their families |
| Postvention | |
| Support for families bereaved by suicide | Not reported |
| Other postventions | Not reported |
| Guideline’s rationale for including family-based intervention(s) in recommendations | |
| Family as a risk factor | Not reported |
| Family as a protective factor | Not reported |
| Other | Not reported |
| Guideline’s measures of implementation and effectiveness of family-based intervention(s) | |
| Measures of Implementation | - Development and approval of regional plans to implement a set of measures to prevention of suicidal behaviour in Belarus - Organization of structure of regional healthcare facilities, such as hospitals, to be able to support suicide prevention strategies |
| Measures of Effectiveness | - Organization, coordination, and control of the leaders of council, regional chairmen, and regional administrations increase effectiveness of the strategies |
| Inclusion of action plan or progress reported | Not reported |
| Rate of suicide from WHO | 26.2 per 100 000 |
| Belgium^51^ | |
| Year | 2012-2020 |
| Title | Flemish action plan for prevention of suicide |
| Population of Interest | General |
| Family Strategy(ies) | Yes |
| Prevention | |
| Suicide-related awareness, education, psychoeducation for families | Not reported |
| Increase familial resilience | - Forming formal networks around elderly; a method of personal planning to help build bridges back to family |
| Family-based psychosocial counselling | Not reported |
| Other preventions | - Strengthen parents in their supporting role in order to increase self-confidence and self-effectiveness, especially when assessing warning signs in children |
| Intervention | |
| Acute family-related intervention strategies | Not reported |
| Other interventions | Not reported |
| Postvention | |
| Support for families bereaved by suicide | - Collaboration with the government to provide supports for those bereaved by suicide |
| Other postventions | Not reported |
| Guideline’s rationale for including family-based intervention(s) in recommendations | |
| Family as a risk factor | Not reported |
| Family as a protective factor | Not reported |
| Other | Not reported |
| Guideline’s measures of implementation and effectiveness of family-based intervention(s) | |
| Measures of Implementation | - Development of communication plan between all organizations and ministries involved in intervention, and establishment a clear timeline |
| Measures of Effectiveness | Scientific basis used to assess interventions presented in new research |
| Inclusion of action plan or progress reported | Not reported |
| Rate of suicide from WHO | 20.7 per 100 000 |
| Bulgaria^34^ | |
| Year | 2013-2018 |
| Title | National program of suicide prevention in Bulgaria |
| Population of Interest | General |
| Family Strategy(ies) | Yes |
| Prevention | |
| Suicide-related awareness, education, psychoeducation for families | - Strengthen familial tolerance of parasuicide related events |
| Increase familial resilience | Not reported |
| Family-based psychosocial counselling | Not reported |
| Other preventions | Not reported |
| Intervention | |
| Acute family-related intervention strategies | Not reported |
| Other interventions | Not reported |
| Postvention | |
| Support for families bereaved by suicide | Not reported |
| Other postventions | Not reported |
| Guideline’s rationale for including family-based intervention(s) in recommendations | |
| Family as a risk factor | Not reported |
| Family as a protective factor | Not reported |
| Other | Not reported |
| Guideline’s measures of implementation and effectiveness of family-based intervention(s) | |
| Measures of Implementation | - Work with mass media at local and national levels, develop a training program for journalists, establish public councils and coalitions at the district level, promote international cooperation |
| Measures of Effectiveness | - Information system of mandatory reporting of suicidal acts adds to data collection |
| Inclusion of action plan or progress reported | - 2013-2018 action plan from Bulgarian government presents timeline and associated actions that will be carried out; actions are derived from the national program^52^ |
| Rate of suicide from WHO | 11.5 per 100 000 |
| Croatia^63^ | |
| Year | 2011-2013 |
| Title | Suicide prevention program in children and young people |
| Population of Interest | Children and youth |
| Family Strategy(ies) | Yes |
| Prevention | |
| Suicide-related awareness, education, psychoeducation for families | - Psychoeducation and counseling in the area of the development of parenting and communication |
| Increase familial resilience | Not reported |
| Family-based psychosocial counselling | - Development of guidelines and counselling for children and young people from families that have history of mental disorder |
| Other preventions | - Programs that encourage early interaction between parent and child |
| Intervention | |
| Acute family-related intervention strategies | Not reported |
| Other interventions | Not reported |
| Postvention | |
| Support for families bereaved by suicide | Not reported |
| Other postventions | Not reported |
| Guideline’s rationale for including family-based intervention(s) in recommendations | |
| Family as a risk factor | - An analysis conducted in 2005 showed that the boys who tried suicide more often take marijuana, less satisfied with family finances situation, they are more prone to aggressive and delinquent behavior and thinking about self-harm |
| Family as a protective factor | Not reported |
| Other | - The healthy mental development of children and young people is based on both biological preconditions and on the relationship of family and environment in suicide risk |
| Guideline’s measures of implementation and effectiveness of family-based intervention(s) | |
| Measures of Implementation | - Implementing authority: Ministry of Science, Education and Sports; Ministry of Health and Social Welfare; Ministry of the Family, Veterans' Affairs and Intergenerational Solidarity - Implementation period: 2011-2013 - Implementation indicators   - Prepared records of existing education and counseling programs   - Developed guidelines for the development of additional education and counseling programs |
| Measures of Effectiveness | - Monitoring the availability of experts and services in the field of mental health protection of children and young people in the health and social care, education, family protection and non-governmental sectors organization |
| Inclusion of action plan or progress reported | Not reported |
| Rate of suicide from WHO | 16.5 per 100 000 |
| Denmark^29^ | |
| Year | 2006 |
| Title | Suicide prevention in children and adolescents |
| Population of Interest | Children and adolescents in all municipalities |
| Family Strategy(ies) | Yes |
| Prevention | |
| Suicide-related awareness, education, psychoeducation for families | Not reported |
| Increase familial resilience | Not reported |
| Family-based psychosocial counselling | Not reported |
| Other preventions | Not reported |
| Intervention | |
| Acute family-related intervention strategies | - Increased suicide risk should involve immediate involvement of the child’s close family network such as parents |
| Other interventions | - All interventions should always try to involve the parents when the young person experiences dissatisfaction and suicidal behaviour - Parents and children work with healthcare providers to plan for solutions and coordinate the various initiatives |
| Postvention | |
| Support for families bereaved by suicide | - Long-term follow-up, gradually spread over time, with bereaved family members should occur, especially if mental illness is present in surviving family members |
| Other postventions | Not reported |
| Guideline’s rationale for including family-based intervention(s) in recommendations | |
| Family as a risk factor | - Care failure where suicidal children and adolescents have been subjected to mental and physical violence, lack of support and care in the home over time, often as a result of weakened parental care   - May be as a result of mental illness in the parents - Death of parents, parents divorce as risk factors |
| Family as a protective factor | - Stress and life stressors are managed by close family networks |
| Other | Not reported |
| Guideline’s measures of implementation and effectiveness of family-based intervention(s) | |
| Measures of Implementation | - Dissemination of knowledge and training to the municipality, citizens and partners to be informed about the emergency preparedness and strategies |
| Measures of Effectiveness | Not reported |
| Inclusion of action plan or progress reported | Not reported |
| Rate of suicide from WHO | 12.8 per 100 000 |
| England^92^ | |
| Year | 2012 |
| Title | Preventing suicide in England |
| Population of Interest | General |
| Family Strategy(ies) | Yes |
| Prevention | |
| Suicide-related awareness, education, psychoeducation for families | Not reported |
| Increase familial resilience | Not reported |
| Family-based psychosocial counselling | - Extension of psychological therapies for children to also be for their families |
| Other preventions | - The healthy child program focuses on health promotion and early intervention with families |
| Intervention | |
| Acute family-related intervention strategies | Not reported |
| Other interventions | Not reported |
| Postvention | |
| Support for families bereaved by suicide | - The strategy draws on the National Suicide Prevention Strategy Advisory Group, some of whom have been bereaved by suicide within their families - Provide effective and timely emotional and practical support for families bereaved by suicide - Have local responses in place during the aftermath of a suicide, and provide information to family - INQUEST Charity has a handbook to help specialists deal with bereaved families in the context of prison custody - The compassionate friends program supports bereaved children and their parents |
| Other postventions | Not reported |
| Guideline’s rationale for including family-based intervention(s) in recommendations | |
| Family as a risk factor | - Links between mental illness and family mental illness, divorce, family breakdown, family bereavement, especially for children but also for adults |
| Family as a protective factor | Not reported |
| Other | - Very significant difficulties for family members and friends in recognizing and responding to suicide |
| Guideline’s measures of implementation and effectiveness of family-based intervention(s) | |
| Measures of Implementation | - The Department of Health has recently made a grant to Survivors of Bereavement by suicide to help support families and friends of those affected |
| Measures of Effectiveness | - Local data collection and suicide audits through projects such as real-time surveillance of suicides - Exploration of the innovative use of technology and predictive analytics to identify those most at risk of suicide |
| Inclusion of action plan or progress reported | - In 2010 a Cross Government Suicide Prevention Plan was published to provide timelines and implementation, surveillance indicators for the strategy’s outlines in the 2012 document^35^ |
| Rate of suicide from WHO | 8.9 per 100 000 |
| Finland ^30^ | |
| Year | 2020-2030 |
| Title | National Mental Health Strategy and Programme for Suicide Prevention |
| Population of Interest | General |
| Family Strategy(ies) | Yes |
| Prevention | |
| Suicide-related awareness, education, psychoeducation for families | - Supporting children’s and families’ competence in the digital environment |
| Increase familial resilience | Not reported |
| Family-based psychosocial counselling | Not reported |
| Other preventions | - Restoration of practical help for families will be created via legislative processes to reduce poverty in families and support parenting - Early intervention for people in crises or difficult life circumstances may prevent further suicides - Development of a more family friendly working life to allow parents to be more attentive to children - Broad based health services required to take client and the family into account when providing supportive services |
| Intervention | |
| Acute family-related intervention strategies | - Providing people at immediate risk of suicide with urgent psychiatric consultation to assess treatment need and developing a treatment plan which includes safety planning if necessary |
| Other interventions | Not reported |
| Postvention | |
| Support for families bereaved by suicide | - Prioritization of treatment for people at risk of suicide and providing increased support for bereaved friends and family |
| Other postventions | Not reported |
| Guideline’s rationale for including family-based intervention(s) in recommendations | |
| Family as a risk factor | - Families in living in poor socioeconomic circumstances have a higher risk of depression are more likely to experiment with substance use   - This is linked to increased risk of mental health difficulties and lower educational attainment |
| Family as a protective factor | - Support of friends and family listed as protective factors - Grandparents and other close adults may be beneficial for a child’s development and the family’s wellbeing |
| Other | - Mental health is a form of capital for individuals, families, communities and society as a whole which can be looked after and invested in; it can be promoted by supporting healthy development in early childhood education promoting wellbeing at work, and through access to safe surroundings. |
| Guideline’s measures of implementation and effectiveness of family-based intervention(s) | |
| Measures of Implementation | - The objectives will be implemented by increasing the availability of preventive services and therapies at the basic level and by improving the collaboration of bodies that establish services |
| Measures of Effectiveness | - Long-tern goal-oriented action based on effective interventions, research, and sufficient resources |
| Inclusion of action plan or progress reported | Not reported |
| Rate of suicide from WHO | 15.9 per 100 000 |
| France ^53^ | |
| Year | 2011-2014 |
| Title | National program against suicide |
| Population of Interest | General |
| Family Strategy(ies) | Yes |
| Prevention | |
| Suicide-related awareness, education, psychoeducation for families | Not reported |
| Increase familial resilience | Not reported |
| Family-based psychosocial counselling | Not reported |
| Other preventions | Not reported |
| Intervention | |
| Acute family-related intervention strategies | - Emergency physicians trained to relay information to and support families |
| Other interventions | Not reported |
| Postvention | |
| Support for families bereaved by suicide | - Postvention focuses on better support for families and loved ones targeted by training of professionals |
| Other postventions | Not reported |
| Guideline’s rationale for including family-based intervention(s) in recommendations | |
| Family as a risk factor | Not reported |
| Family as a protective factor | Not reported |
| Other | Not reported |
| Guideline’s measures of implementation and effectiveness of family-based intervention(s) | |
| Measures of Implementation | - National Suicide Prevention Union and National Institute for Prevention and Health Education collaborate to train and evaluate interventions |
| Measures of Effectiveness | - Carry out new studies and research to improve knowledge - Implement specific modalities for monitoring patients to prevent relapse |
| Inclusion of action plan or progress reported | Not reported |
| Rate of suicide from WHO | 17.7 per 100 000 |
| Ireland^90^ | |
| Year | 2015-2020 |
| Title | Ireland’s National Strategy to Reduce Suicide |
| Population of Interest | General |
| Family Strategy(ies) | Yes |
| Prevention | |
| Suicide-related awareness, education, psychoeducation for families | - Ensure that accurate information and guidance on effective suicide prevention are provided for community-based organisations (e.g. family resource centres) - Increased standardisation of approach for service delivery, through dissemination of guidelines and protocols to communities and families - Expand areas of engagement in working with disadvantaged families and communities |
| Increase familial resilience | Not reported |
| Family-based psychosocial counselling | Not reported |
| Other preventions | Not reported |
| Intervention | |
| Acute family-related intervention strategies | Not reported |
| Other interventions | Not reported |
| Postvention | |
| Support for families bereaved by suicide | - Improve the uniformity, effectiveness and timeliness of support services to families and communities bereaved by suicide - Deliver enhanced bereavement support services, such as counselling, to families and communities that are impacted by suicide |
| Other postventions | Not reported |
| Guideline’s rationale for including family-based intervention(s) in recommendations | |
| Family as a risk factor | - Family history of suicide, familial psychiatric problems presented as a risk factor - Social exclusion is fueled by familial problems and conflict, often presenting as a risk factor for suicide |
| Family as a protective factor | - Strong familial ties presented as protective factor |
| Other | Not reported |
| Guideline’s measures of implementation and effectiveness of family-based intervention(s) | |
| Measures of Implementation | - Strong working relationship with multiple organizations including health and wellbeing based sectors |
| Measures of Effectiveness | - Policies and interventions are evaluated based on evidence |
| Inclusion of action plan or progress reported | Not reported |
| Rate of suicide from WHO | 11.5 per 100 000 |
| Italy^59^ | |
| Year | 2017 |
| Title | National plan for the prevention of suicidal conduct in the penitentiary system for adults |
| Population of Interest | Adults in penitentiary system |
| Family Strategy(ies) | Yes |
| Prevention | |
| Suicide-related awareness, education, psychoeducation for families | Not reported |
| Increase familial resilience | Not reported |
| Family-based psychosocial counselling | Not reported |
| Other preventions | - Create the opportunity for family members to express concern a suicide risk - Follow-up/contact with detained relative to verify concerns - All contact must be regulated and following certain procedures, and any identified risk must be reported - Family must be given the opportunity to report doubts about emotion and psychological risk identified during interviews, to staff |
| Intervention | |
| Acute family-related intervention strategies | Not reported |
| Other interventions | Not reported |
| Postvention | |
| Support for families bereaved by suicide | Not reported |
| Other postventions | Not reported |
| Guideline’s rationale for including family-based intervention(s) in recommendations | |
| Family as a risk factor | Not reported |
| Family as a protective factor | - Protective factors (social support, family , stable romantic relationship, good skills adaptation to the environment, etc.) |
| Other | Not reported |
| Guideline’s measures of implementation and effectiveness of family-based intervention(s) | |
| Measures of Implementation | - Collaboration and agreement between various ministries in order to implement strategies |
| Measures of Effectiveness | - Risk testing and periodic testing in order to test for effectiveness of recommendations |
| Inclusion of action plan or progress reported | Not reported |
| Rate of suicide from WHO | 8.2 per 100 000 |
| Italy^70^ | |
| Year | 2017 |
| Title | National plan^1,2^ ^1,2^for the preventi^1,2^on o^1,2^f self-harm and suicidal risk in residential juvenile services: Department for Juvenile and Community Justice |
| Population of Interest | Adults in penitentiary system |
| Family Strategy(ies) | Yes |
| Prevention | |
| Suicide-related awareness, education, psychoeducation for families | Not reported |
| Increase familial resilience | Not reported |
| Family-based psychosocial counselling | Not reported |
| Other preventions | - Monitor family interviews over the phone or in person to assess the level of familial problems present - For minors and children who are not able to meet with parents, video calls will be set up - Strengthen family collaboration with social services to create a plan for reintegration into society - Multidisciplinary team should involve family wherever possible |
| Intervention | |
| Acute family-related intervention strategies | Not reported |
| Other interventions | Not reported |
| Postvention | |
| Support for families bereaved by suicide | Not reported |
| Other postventions | Not reported |
| Guideline’s rationale for including family-based intervention(s) in recommendations | |
| Family as a risk factor | - Critical family distress (separation, violence suffered, fear of abandonment) - Absence of communication with family is considered a risk factor |
| Family as a protective factor | - Protective factors (social support, family , stable romantic relationship, good skills adaptation to the environment, etc.) |
| Other | - Need to consider family in psycho-social aspects in children development |
| Guideline’s measures of implementation and effectiveness of family-based^1–8^ intervention(s) | |
| Measures of Implementation | - Collaboration and agreement between various ministries in order to implement strategies locally |
| Measures of Effectiveness | - Risk testing and periodic testing in order to test for effectiveness of recommendations |
| Inclusion of action plan or progress reported | Not reported |
| Rate of suicide from WHO | 8.2 per 100 000 |
| Lithuania ^42^ | |
| Year | 2017 |
| Title | Suicide prevention and assistance for persons with suicide risk |
| Population of Interest | General |
| Family Strategy(ies) | Yes |
| Prevention | |
| Suicide-related awareness, education, psychoeducation for families | Not reported |
| Increase familial resilience | Not reported |
| Family-based psychosocial counselling | Not reported |
| Other preventions | - Promote childcare and adoption in families - Establishment of services to provide support for victims of familial violence |
| Intervention | |
| Acute family-related intervention strategies | Not reported |
| Other interventions | Not reported |
| Postvention | |
| Support for families bereaved by suicide | Not reported |
| Other postventions | Not reported |
| Guideline’s rationale for including family-based intervention(s) in recommendations | |
| Family as a risk factor | - Family suicide decreases resilience and increases vulnerability |
| Family as a protective factor | Not reported |
| Other | - Childcare and adoption are directly linked to creating a safe environment |
| Guideline’s measures of implementation and effectiveness of family-based intervention(s) | |
| Measures of Implementation | - Audits completed to ensure interventions are being administered |
| Measures of Effectiveness | - Implementation of suicide interventions based on research acquired evidence and reliable data |
| Inclusion of action plan or progress reported | During the audited period, the Ministry of Health suicide prevention measures planned to reduce health inequalities in Lithuania in 2014–2020 |
| Rate of suicide from WHO | 31.9 per 100 000 |
| Luxembourg^43^ | |
| Year | 2015-2019 |
| Title | Luxembourg national suicide prevention plan |
| Population of Interest | General |
| Family Strategy(ies) | Yes |
| Prevention | |
| Suicide-related awareness, education, psychoeducation for families | - Multifactorial interventions that include family, reduce access to means of suicide, increase awareness of the prevention of suicide, promote solidarity in family   - Education for identification of symptoms   - Create visible and accessible services |
| Increase familial resilience | - Build resilience, create an environment that promotes self-help and demand help, provide support structures support - Promote environments (families, schools) to minimize emotions (anxiety, stress, sadness, grief, etc.) without stigmatization |
| Family-based psychosocial counselling | Not reported |
| Other preventions | Not reported |
| Intervention | |
| Acute family-related intervention strategies | - Develop support procedures for people who have attempted suicide and for members of their families |
| Other interventions | Not reported |
| Postvention | |
| Support for families bereaved by suicide | Not reported |
| Other postventions | Not reported |
| Guideline’s rationale for including family-based intervention(s) in recommendations | |
| Family as a risk factor | - Risk of suicide or suicidal behavior: mental illness in family, substance abuse problems among parents, childhood abuse, early abandonment and losses, isolation, lack of ties significant in the family, divorce, death in family |
| Family as a protective factor | - Family connectedness is a protective factor |
| Other | Not reported |
| Guideline’s measures of implementation and effectiveness of family-based intervention(s) | |
| Measures of Implementation | - Guideline has results and achievemen^1–4^t indicators for specific objectives |
| Measures of Effectiveness | - Number of recommendation guides disseminated in the event of a crisis to families - Number of leaflets distributed listing the useful addresses for a caring for the family after a suicide or attempted suicide |
| Inclusion of action plan or progress reported | - Pragmatic action plan to determine whether strategies have had the desired impact |
| Rate of suicide from WHO | 13.5 per 100 000 |
| Netherlands^22^ | |
| Year | 2007 |
| Title | Reducing suicide: policy advice |
| Population of Interest | General |
| Family Strategy(ies) | Yes |
| Prevention | |
| Suicide-related awareness, education, psychoeducation for families | - For family members and those directly involved in patients with psychiatric problems psychoeducational courses and programs are employed by task groups   - Participants learn how to take care of themselves, prevent them from becoming overloaded |
| Increase familial resilience | Not reported |
| Family-based psychosocial counselling | - Mother-baby intervention where the family participates in meetings with the children to talk about familial psychiatric problems |
| Other preventions | Not reported |
| Intervention | |
| Acute family-related intervention strategies | Not reported |
| Other interventions | Not reported |
| Postvention | |
| Support for families bereaved by suicide | - Support for survivors and loved ones by providing attention, especially for children. - Emergency workers are trained on cognitive behavioral therapy and psychoeducation to positively influence the course of grief after suicide |
| Other postventions | Not reported |
| Guideline’s rationale for including family-based intervention(s) in recommendations | |
| Family as a risk factor | - Factors such as sexual abuse, physical abuse and emotional neglect by parents, long absence of one or both parents, divorce from parents, long-term psychopathology in parents associated with suicidal behavior in adolescents; - Suicidal behavior is often preceded by stressful exposure or negative life events associated with shame, humiliation, embarrassment groin, failure or threat (e.g. interpersonal conflicts, marriage or relationship problems, family disputes, unemployment, relocation and retirement) - Personality traits such as hopelessness, neuroticism, anxiety, cognitive rigidity, worry, impulsivity and aggression are also risk factors for suicide |
| Family as a protective factor | - Involved responsiveness and care are important protective factors - Protective factors relationships: including quality of parenting, close relationship with adults (parents, family, teachers), contacts with peers |
| Other | Not reported |
| Guideline’s measures of implementation and effectiveness of family-based intervention(s) | |
| Measures of Implementation | Not reported |
| Measures of Effectiveness | - Policy indicates for each recommendation what the scientific status (evidence, consensus or assumption) is - It also indicates the execution of the term, costs and source of funding |
| Inclusion of action plan or progress reported | - National suicide prevention agenda 2018-2021 outlines timelines and progress of the strategy^1,232^ |
| Rate of suicide from WHO | 12.6 per 100 000 |
| Northern Ireland ^68^ | |
| Year | 2019-2024 |
| Title | Protect Life 2: A Strategy for Preventing Suicide and Self Harm in Northern Ireland 2019-2024 |
| Population of Interest | General |
| Family Strategy(ies) | Yes |
| Prevention | |
| Suicide-related awareness, education, psychoeducation for families | - Facebook linked to a suicide prevention tool to outline advice, resources and support to people with suicidal thoughts and their families - Increased contact and better education for families of people deemed to be at risk |
| Increase familial resilience | Not reported |
| Family-based psychosocial counselling | Not reported |
| Other preventions | Not reported |
| Intervention | |
| Acute family-related intervention strategies | Not reported |
| Other interventions | Not reported |
| Postvention | |
| Support for families bereaved by suicide | - Health and Social Care Trusts provide suicide prevention co-ordinators such as family liaison officers (who work with bereaved families) - The regional Mental Health Care Pathway “*You in Mind*” commits to taking a recovery approach, which involves the family promoting recovery and safety through a personal safety plan - Referral process for family members where death by suicide occurred later in hospital - Development of standardised guidance for suicide bereaved families in the primary care setting - Representatives of bereaved families give families encouragement to continue to develop networks |
| Other postventions | Not reported |
| Guideline’s rationale for including family-based intervention(s) in recommendations | |
| Family as a risk factor | - Family discord as a risk factor for children and youth suicide - Families experience isolation, high responsibility, and lack of knowledge when caring for a person at risk of suicide |
| Family as a protective factor | - Developing family and community connectedness listed as a protective factor |
| Other | Not reported |
| Guideline’s measures of implementation and effectiveness of family-based intervention(s) | |
| Measures of Implementation | - Public and private sector organisations, academia, carers, voluntary & community agencies, and groups representing bereaved families - Public Health Agency and Trusts responsible for implementation |
| Measures of Effectiveness | - Performance reports will be published outlining a summary of performance of the actions in the action plan - An evaluation framework developed to critically examine the outcome and impact of interventions and programmes against the strategy objectives; specific outcome indicators will be created |
| Inclusion of action plan or progress reported | - Public health agency will develop action/implementation plan to monitor progress in implementation of the strategies |
| Rate of suicide from WHO | Not reported |
| Norway^57^ | |
| Year | 2020-2025 |
| Title | Action plan for suicide prevention: no one to lose |
| Population of Interest | General |
| Family Strategy(ies) | Yes |
| Prevention | |
| Suicide-related awareness, education, psychoeducation for families | - Government provides information to parents about exposure of children and adolescents to harmful content on the internet - The government has developed a family module that teaches families how to take care of relatives better |
| Increase familial resilience | Not reported |
| Family-based psychosocial counselling | Not reported |
| Other preventions | - Children, youth and the family agencies have a number of measures to prevent suicide and self-harm - Veterans organizations offer volunteers that families can contact for help and personal work |
| Intervention | |
| Acute family-related intervention strategies | Not reported |
| Other interventions | Not reported |
| Postvention | |
| Support for families bereaved by suicide | - Family protection service can offer conversations with children bereaved by suicide, and to adult survivors if a child or youth have taken their lives |
| Other postventions | - Immediate and long-term follow-up is also suicide prevention as suicide survivors have an increased risk of taking their own lives |
| Guideline’s rationale for including family-based intervention(s) in recommendations | |
| Family as a risk factor | Not reported |
| Family as a protective factor | Not reported |
| Other | - Many people feel that they do not get necessary follow-up, and that they are unable to cope with school or work - There is a lack of studies on health service use and follow-up of survivors by suicide |
| Guideline’s measures of implementation and effectiveness of family-based intervention(s) | |
| Measures of Implementation | - The government will implement regional public awareness campaigns with national superstructure about suicide prevention |
| Measures of Effectiveness | Not reported |
| Inclusion of action plan or progress reported | Not reported |
| Rate of suicide from WHO | 12.2 per 100 000 |
| Portugal^76^ | |
| Year | 2013-2017 |
| Title | National suicide prevention plan |
| Population of Interest | General |
| Family Strategy(ies) | Yes |
| Prevention | |
| Suicide-related awareness, education, psychoeducation for families | Not reported |
| Increase familial resilience | - Family support programs increase resilience |
| Family-based psychosocial counselling | - If family members are also suffering from psychiatric pathology, they should be involved in treatment, seeking to create healthy alternatives |
| Other preventions | - Prevention campaigns in schools create synergies between school, family, health services and the community, favoring an ecological and integrative vision - Campaigns connect LGBT individuals with family networks - Facilitation of access to health care when vulnerabilities are identified such as family problems, loss of housing |
| Intervention | |
| Acute family-related intervention strategies | Not reported |
| Other interventions | Not reported |
| Postvention | |
| Support for families bereaved by suicide | - The post-intervention strategy aims to support family of a person who committed suicide, to facilitate the resolution of the grieving process, but also to prevent other suicidal acts |
| Other postventions | Not reported |
| Guideline’s rationale for including family-based intervention(s) in recommendations | |
| Family as a risk factor | - Family history of suicide, familial violence, abuse physical or sexual and family neglect increase the risk of suicide - Family unemployment or financial problems are also risk factors for suicide |
| Family as a protective factor | - Feeling of belonging to a family, to a school, to a group, to an institution or community, can protect a young person from suicide |
| Other | - Programs focused on families have a greater impact than those focused on exclusively in individuals - policies are most effective when accompanied by individual interventions and family members - Community programs should include standards that support aid and well-being in family, work, school - Familial survivors and individuals who carry out suicidal acts are often stigmatized - In some cultures or religious groups, suicide can be tolerated in a context such as suicides in defense of the honor of himself or his family - Divorce insufficient family and social support, have a major impact on the isolation of seniors |
| Guideline’s measures of implementation and effectiveness of family-based intervention(s) | |
| Measures of Implementation | - Due to previous difficulty in implementation and effectiveness, they outlined these measures:   - Prevention programs must be designed, not just to promote protective factors, but also to reverse or reduce factors known risk factors   - Programs focused on families have a greater impact than those focused on exclusively in individuals |
| Measures of Effectiveness | - The monitoring and evaluation of the plan will allow its adequacy and redefinition whenever necessary. |
| Inclusion of action plan or progress reported | Not reported |
| Rate of suicide from WHO | 14.0 per 100 000 |
| Scotland^93^ | |
| Year | 2013-2016 |
| Title | Suicide Prevention Strategy |
| Population of Interest | General |
| Family Strategy(ies) | Yes |
| Prevention | |
| Suicide-related awareness, education, psychoeducation for families | - National and local campaigns should focus on breaking down stigma and common misconceptions around suicide and encouraging families to talk about feelings with family |
| Increase familial resilience | Not reported |
| Family-based psychosocial counselling | Not reported |
| Other preventions | Not reported |
| Intervention | |
| Acute family-related intervention strategies | Not reported |
| Other interventions | - Health societies for Scotland will work together to develop and extend the current approach of workforce development activity to address a wider range of experience and in a wider range of contexts including families and communities |
| Postvention | |
| Support for families bereaved by suicide | - Multiple resources listed for families bereaved by suicide |
| Other postventions | Not reported |
| Guideline’s rationale for including family-based intervention(s) in recommendations | |
| Family as a risk factor | Not reported |
| Family as a protective factor | Not reported |
| Other | Not reported |
| Guideline’s measures of implementation and effectiveness of family-based intervention(s) | |
| Measures of Implementation | - The Scottish Government has set up and fund a National Suicide Prevention Leadership Group (NSPLG) in September 2018 that will make recommendations on supporting the development and delivery of local action plans ^1^backed by the parliament |
| Measures of Effectiveness | - NSPLG will use evidence on the effectiveness of differing models of crisis support to make recommendations to service providers |
| Inclusion of action plan or progress reported | - Outlined in the 2018-2022 the Scottish Government will fund the creation and implementation suicide prevention training and the NSPLG will support dissemination and implementation across public and private sectors^36^ |
| Rate of suicide from WHO | Not reported |
| Spain^28^ | |
| Year | 2006 |
| Title | Facing the reality of suicide |
| Population of Interest | General |
| Family Strategy(ies) | Yes |
| Prevention | |
| Suicide-related awareness, education, psychoeducation for families | Family school program:   - Provide families with information on mental health and training - Provide families with psychoeducation, family respite |
| Increase familial resilience | Not reported |
| Family-based psychosocial counselling | Not reported |
| Other preventions | Family school program:   - Promote family communication skills, recovery of family roles - Reduce stressful situations and family overload - Encourage problem solving within the family - Breaking the social isolation and stigma situation of families of people with mental illness |
| Intervention | |
| Acute family-related intervention strategies | - If a family member is at risk 1) Don't promise confidentiality. Seek help also between your family and friends 2) Seek professional help and report any family history of suicide - Family member should be approached to talk to, if person is having thoughts of suicide - Develop a security plan with your family member to address their safety |
| Other interventions | Not reported |
| Postvention | |
| Support for families bereaved by suicide | Not reported |
| Other postventions | Not reported |
| Guideline’s rationale for including family-based intervention(s) in recommendations | |
| Family as a risk factor | - Family history of suicide: there are doubts about if the relationship between this factor and suicide risk is due to genetic factors or the social environment - People with bipolar disorder suicide generally occurs when work, study, family or emotional pressures are very big |
| Family as a protective factor | Not reported |
| Other | Not reported |
| Guideline’s measures of implementation and effectiveness of family-based intervention(s) | |
| Measures of Implementation | - The guide is presented as a prevention tool itself. It is meant for healthcare professionals, national initiatives, and for the individual at risk of suicide; it does not include an action plan |
| Measures of Effectiveness | Not reported |
| Inclusion of action plan or progress reported | Not reported |
| Rate of suicide from WHO | 8.7 per 100 000 |
| Sweden^44(p8)^ | |
| Year | 2008 |
| Title | National action programme for suicide prevention |
| Population of Interest | General |
| Family Strategy(ies) | Yes |
| Prevention | |
| Suicide-related awareness, education, psychoeducation for families | - Raise skill levels among family and other key individuals in the care services by providing training to identify suicide problems |
| Increase familial resilience | Not reported |
| Family-based psychosocial counselling | Not reported |
| Other preventions | - Support voluntary organizations: Important that multi public body groups work with survivor representatives service­ users but the working methods must not pose obstacles for participating patients and families |
| Intervention | |
| Acute family-related intervention strategies | Not reported |
| Other interventions | Not reported |
| Postvention | |
| Support for families bereaved by suicide | Not reported |
| Other postventions | Not reported |
| Guideline’s rationale for including family-based intervention(s) in recommendations | |
| Family as a risk factor | Not reported |
| Family as a protective factor | Not reported |
| Other | Not reported |
| Guideline’s measures of implementation and effectiveness of family-based intervention(s) | |
| Measures of Implementation | - Broad collaboration between the Swedish government and the Swedish Parliament, authorities, municipalities and county councils, universities and colleges, voluntary organisations and organisations that support bereaved families is needed for implementation |
| Measures of Effectiveness | - Authorities, universities and research and development units have the task of obtaining, compiling and distributing knowledge within their area of activity - Event analysis after suicide is performed in order to find out how and why a suicide occurred, and what action may be taken to ensure it does not happen again |
| Inclusion of action plan or progress reported | - The National Institute of Public Health's and the National Board of Health and Welfare's proposal for a national program (action plan) is in action |
| Rate of suicide from WHO | 14.8 per 100 000 |
| Switzerland^46^ | |
| Year | 2016 |
| Title | Suicide prevention in Switzerland: starting point, need for action and action plan |
| Population of Interest | General |
| Family Strategy(ies) | Yes |
| Prevention | |
| Suicide-related awareness, education, psychoeducation for families | - Relatives, friends and professionals who are affected by a suicide have access to support services to help them cope |
| Increase familial resilience | Not reported |
| Family-based psychosocial counselling | - Telephone counselling and emergency services should be tailored to the needs of those affected as well as their family and friends |
| Other preventions | Not reported |
| Intervention | |
| Acute family-related intervention strategies | Not reported |
| Other interventions | Not reported |
| Postvention | |
| Support for families bereaved by suicide | - Establishment of support services for bereaved relatives, and the notification of available services such as emergency psychological support and self help groups |
| Other postventions | Not reported |
| Guideline’s rationale for including family-based intervention(s) in recommendations | |
| Family as a risk factor | - Suicides in family context are also a risk factor for suicidal behaviours, where up to ten relatives or friends experience great mental stress with each suicide |
| Family as a protective factor | Not reported |
| Other | - Suicide causes great suffering for surviving relatives |
| Guideline’s measures of implementation and effectiveness of family-based intervention(s) | |
| Measures of Implementation | - Collaboration between difference sectors will increase feasibility and implementation |
| Measures of Effectiveness | - Action plan has 10 goals and 19 key measures that have relevant scientific background and data required to be managed and evaluated - Services routinely collect quantitative data that enable the management and evaluation of interventions - Closure of knowledge gaps regarding primary, secondary and tertiary suicide prevention with qualitative and quantitative research |
| Inclusion of action plan or progress reported | - This document itself is an action plan for Switzerland |
| Rate of suicide from WHO | 17.2 per 100 000 |
| Supplemental table 4.0: Guidelines identified from Africa | |
| Namibia^87^ | |
| Year | 2012-2016 |
| Title | National strategic plan on the prevention of suicide in Namibia |
| Population of Interest | General |
| Family Strategy(ies) | Yes |
| Prevention | |
| Suicide-related awareness, education, psychoeducation for families | - Suggesting that people engage with family and friends, and giving information on things like help line services - Helping people to be able to identify risk in friends and family |
| Increase familial resilience | Not reported |
| Family-based psychosocial counselling | - Promote positive connectedness between individuals and family |
| Other preventions | Not reported |
| Intervention | |
| Acute family-related intervention strategies | - Family members and friends are best placed to observe declines, and need to be both informed of referral options and enabled in suggesting them - Relying on family, friends, local leaders and religious leaders to help counsel people is an important first step, but second steps should be to engage with trained service providers or support networks (e.g., suicide survivors) |
| Other interventions | Not reported |
| Postvention | |
| Support for families bereaved by suicide | Not reported |
| Other postventions | Not reported |
| Guideline’s rationale for including family-based intervention(s) in recommendations | |
| Family as a risk factor | - Romantic relationships and families are often catalysts for suicide attempts, the model needs to consider these risks when developing a response |
| Family as a protective factor | - Original model places people and their immediate families at the centre and kinship, community around it |
| Other | - Qualitative findings suggest that, even when family, friends and religious leaders are sympathetic; they lack the skills to be able to offer the support - Only one-third of female respondents and one-quarter of male respondents who had attempted suicide, had gone to a service provider (e.g. family) for counselling and support after the attempt |
| Guideline’s measures of implementation and effectiveness of family-based intervention(s) | |
| Measures of Implementation | - % of objectives achieved/implemented; review meetings for initiatives |
| Measures of Effectiveness | - Cost effectiveness and budget outline for the evaluation of strategy value |
| Inclusion of action plan or progress reported | Not reported |
| Rate of suicide from WHO | 8.7 per 100 000 |

Supplemental table 5.0: Guidelines identified from Oceania

| Australia ^91^ | |
| --- | --- |
| Year | 2008; adopted as Australia’s overarching framework in 2011 |
| Title | LIFE: A framework for prevention of suicide in Australia |
| Population of Interest | General |
| Family Strategy(ies) | Yes |
| Prevention | |
| Suicide-related awareness, education, psychoeducation for families | - Symptom identification: Improving understanding of suicide and promoting education for the immediate family, friends, social networks, local doctor, and work colleagues of people at risk |
| Increase familial resilience | - Universal intervention: create stronger, more supportive families and communities |
| Family-based psychosocial counselling | - Longer term treatment: Providing individuals feeling suicidal with ongoing access to supports from family and community, workplace, professional careers and health services |
| Other preventions | - Establishment of safety nets (e.g. family members) when moving between treatment options - Foster environments (e.g. families, where it is acceptable to express emotions (stress, sadness, grief) without a fear of stigma |
| Intervention | |
| Acute family-related intervention strategies | Not reported |
| Other interventions | Not reported |
| Postvention | |
| Support for families bereaved by suicide | - Working with families of those who have taken their own life, to respond to their grief and loss and their elevated risk of suicide |
| Other postventions |  |
| Guideline’s rationale for including family-based intervention(s) in recommendations | |
| Family as a risk factor | - Family discord, and withdrawal from family are presented as risk factors; lack of family support increases vulnerability and potentially the incidence of traumatic occurrences |
| Family as a protective factor | - Family interaction and support listed as a protective factor for suicide as it helps increase resilience |
| Other | Not reported |
| Guideline’s measures of implementation and effectiveness of family-based intervention(s) | |
| Measures of Implementation | - Resource efficiency (staffing, infrastructure, consumption) - Quantity delivered in terms of policy needs, agreed targets, inputs to project |
| Measures of Effectiveness | - Reductions in suicide attempts and/or suicidal thinking - Reductions in risk factors and vulnerabilities to suicidal behaviours - Increase in individual and/or community awareness of appropriate suicide prevention - Changes in behaviours and response to suicide prevention strategies - Improvements in individual protective or resiliency factors |
| Inclusion of action plan or progress reported | - 2017 publishing of implementation plan of national suicide prevention strategy listing specific government and community group timelines to implement evidence-based changes in suicidal behaviour in Australia ^33^ |
| Rate of suicide from WHO | 13.2 per 100 000 |
| Australia^65^ | |
| Year | 2013 |
| Title | National Aboriginal and Torres Strait Islander Suicide Prevention Strategy |
| Population of Interest | Aboriginal and Torres Strait Islander |
| Family Strategy(ies) | Yes |
| Prevention | |
| Suicide-related awareness, education, psychoeducation for families | - Provide information to all parents, families, and young people to help build skills and awareness, to dispel myths and to promote active use of services and supports |
| Increase familial resilience | - Prevention should work across the lifespan, directly with families or with children in schools to ensure that all Aboriginal and Torres Strait Islander children are supported to develop the social and emotional competencies that are the foundations of resilience throughout life |
| Family-based psychosocial counselling | Not reported |
| Other preventions | - Prevention efforts are Undertaken across a range of settings – individual, family, school and community, with multiple components delivered within multiple settings |
| Intervention | |
| Acute family-related intervention strategies | Not reported |
| Other interventions | Not reported |
| Postvention | |
| Support for families bereaved by suicide | - Sensitive postvention and bereavement support for people in peer and family networks - Develop protocols for communication between specialist mental health services and Aboriginal and Torres Strait Islander families regarding intervention needs and support following bereavement |
| Other postventions |  |
| Guideline’s rationale for including family-based intervention(s) in recommendations | |
| Family as a risk factor | - Royal Commission into Aboriginal Deaths in custody (RCIADIC, 1991) drew attention to the links between substance misuse and mental health disorders   - Highlighted the disproportionate number of deaths (over three-quarters) where there was a history of having been forcibly separated from natural families as children - Suicide risk can involve unresolved grief and loss, trauma and abuse, domestic violence, removal from family, substance misuse, family breakdown, cultural dislocation |
| Family as a protective factor | Not reported |
| Other | Not reported |
| Guideline’s measures of implementation and effectiveness of family-based intervention(s) | |
| Measures of Implementation | - Develop local partnerships between existing services such as headspace centres and Aboriginal and Torres Strait Islander community social and emotional wellbeing services |
| Measures of Effectiveness | - Proposed strategies should be implemented in Aboriginal and Torres Strait Islander populations and should be adapted after determining strategy effectiveness |
| Inclusion of action plan or progress reported | - 2017 publishing of implementation plan of national suicide prevention strategy listing specific government and community group timelines to implement evidence-based changes in suicidal behaviour in Australia - 2017 publication of implementation plan includes checkpoints that assess effectiveness at various checkpoints throughout the proposed timeline^33^ |
| Rate of suicide from WHO | 13.2 per 100 000 |
| Aotearoa New Zealand^47^ | |
| Year | Suicide Prevention Strategy 2019–2029 and Suicide Prevention Action Plan 2019–2024 |
| Title | Every Life Matters  He Tapu te Oranga o ia tangata |
| Population of Interest | General population of New Zealand, also with a focus on Maori and Pasifika population |
| Family Strategy(ies) | Yes |
| Prevention | |
| Suicide-related awareness, education, psychoeducation for families | - Develop self-harm prevention resources and guidelines with people with lived experience, for whānau and families, schools and health services - Reorient the existing suicide information service to ensure the right information gets to the people who need it, particularly whānau, families and friends of people at risk or people who have died, and to provide good information to the media |
| Increase familial resilience | - A whānau-centred approach to suicide prevention is about empowering the whānau and family as a whole, rather than focusing on separate individual members and their problems - Work with Māori to identify current whānau, hapū, iwi and community-based wellbeing initiatives that support Māori (particularly tamariki and rangatahi Māori) to connect to their culture and build a strong cultural identity |
| Family-based psychosocial counselling | Not reported |
| Other preventions | - Work with DHBs to develop and implement a range of whānau and family and community- led responses for people experiencing suicidal distress - moving from a largely mental-health- service-based response to enabling communities to nurture and support their whānau and families and community members when they are experiencing suicidal distress - Sharing information between different services removes the need for people to retell their stories of distress and helps them and their families and whānau feel culturally safe - Support Māori whānau, hapū, iwi, Pasifika families and communities to develop solutions to suicide |
| Intervention | |
| Acute family-related intervention strategies | - Train community health and social support services staff, families, whānau, hapū, iwi and community members to identify and support individuals at risk of suicide and refer them to agencies that can help. |
| Other interventions | Not reported |
| Postvention | |
| Support for families bereaved by suicide | - supporting individuals, whānau and families, and communities after a suicide - Expand the Initial Response Service, which provides specialist practical and emotional support to families, whānau and others bereaved by suicide, so that it is available nationwide. |
| Other postventions | Not reported |
| Guideline’s rationale for including family-based intervention(s) in recommendations | |
| Family as a risk factor | Not reported |
| Family as a protective factor | - Protective factors for suicide include good whānau and family relationships |
| Other | - One model of wellbeing is the concept of ‘Te whare tapa wha’, and has four components: taha tinana (physical health), taha hinengaro (mental health), taha whānau (whānau and family health) and taha wairua (spiritual health) |
| Guideline’s measures of implementation and effectiveness of family-based intervention(s) | |
| Measures of Implementation | - The Suicide Prevention Office will work alongside Māori and other agencies and organisations, including district health boards and non-governmental organisations (NGOs) and whānau and families to develop a meaningful and effective monitoring |
| Measures of Effectiveness | - Access to current research, and the sharing of research will identify research gaps - Disseminate information, evidence-informed guidance and research to individuals, whānau and families and communities |
| Inclusion of action plan or progress reported | - **Suicide Prevention Action Plan 2019–2024**identifies specific actions that will to help prevent suicide and support those affected by suicide in Aotearoa New Zealand; same document as strategy |
| Rate of suicide from WHO | 12.1 per 100 000 |
| Cook’s Island^55^ | |
| Year | 2016-2020 |
| Title | Cook Islands National Suicide Prevention Strategy |
| Population of Interest | General |
| Family Strategy(ies) | Yes |
| Prevention | |
| Suicide-related awareness, education, psychoeducation for families | Not reported |
| Increase familial resilience | Not reported |
| Family-based psychosocial counselling | - Support services strengthened to provide family counselling program initiatives |
| Other preventions | - To improve support for all families, especially those socially excluded - Identify, strengthen support services and programs for families that are in crises/at risk - Review, improve and standardize transfer between mental health services settings, guided by the availability of other supports such as family and significant others |
| Intervention | |
| Acute family-related intervention strategies | Not reported |
| Other interventions | Not reported |
| Postvention | |
| Support for families bereaved by suicide | Not reported |
| Other postventions | Not reported |
| Guideline’s rationale for including family-based intervention(s) in recommendations | |
| Family as a risk factor | Not reported |
| Family as a protective factor | Not reported |
| Other | - Premature death from suicide has many adverse consequences, for the family and friends of those who pass away and the wider community |
| Guideline’s measures of implementation and effectiveness of family-based intervention(s) | |
| Measures of Implementation | - Where possible, existing human and other resources will be used to implement the strategy, but where these are not available, new resources will be required and acquired from all sectors |
| Measures of Effectiveness | - Goals include a 50% reduction in the suicide rate by 2020 and a 20% reduction in the suicide attempt rate by 2020 - Yearly progress reports are present on suicide and attempted suicide rates - Research and Development – Ongoing, quality, and multi-disciplinary research will be an essential strand of the strategy and findings will be of greatest value where they can inform and stimulate action and service development |
| Inclusion of action plan or progress reported | Not reported |
| Rate of suicide from WHO | Not reported |
| Fiji^45^ | |
| Year | 2015-2020 |
| Title | Fiji national mental health and suicide prevention policy |
| Population of Interest | General |
| Family Strategy(ies) | Yes |
| Prevention | |
| Suicide-related awareness, education, psychoeducation for families | - Promote the incorporation of Family Life Education into the national school curriculum - Work with health educational authorities to lessen the stigma towards people who have attempted or completed suicide, and their families |
| Increase familial resilience | Not reported |
| Family-based psychosocial counselling | Not reported |
| Other preventions | - People from all sectors, including persons with mental disorders, carers and family members will be engaged in the development and implementation of policies, laws and services relating to mental health and suicide prevention - Improvement of national attitudes towards suicide prevention through local and community workshops focusing on families, workplaces and stress-related lifestyles |
| Intervention | |
| Acute family-related intervention strategies | Not reported |
| Other interventions | Not reported |
| Postvention | |
| Support for families bereaved by suicide | Not reported |
| Other postventions | Not reported |
| Guideline’s rationale for including family-based intervention(s) in recommendations | |
| Family as a risk factor | Not reported |
| Family as a protective factor | Not reported |
| Other | Not reported |
| Guideline’s measures of implementation and effectiveness of family-based intervention(s) | |
| Measures of Implementation | - Clinics and healthcare facilities will be responsible for ensuring that strategies are incorporated into their respective plans and protocols |
| Measures of Effectiveness | - Accurate analysis and research to provide best evidence to suggest the effectiveness of support services for people at risk of suicide and their families - The Committee will report quarterly to the National Mental Health Advisory Council (NMHAC) through the Chair of the National Committee on the Prevention of Suicide |
| Inclusion of action plan or progress reported | - Policies follow the directions of the Mental Health Decree (2010), the Roadmap for Democracy and Sustainable Socio-Economic Development 2009-2014 and the WHO Comprehensive Mental Health Action Plan 2013 – 2020 - Detailed strategies to implement the policy will be found in the National Mental Health and Suicide Prevention Strategic Plan (NMHSPSP) |
| Rate of suicide from WHO | 5.0 per 100 000 |
| Supplemental table 6.0: Guideline identified from South America | |
| Argentina^54^ | |
| Year | 2015 |
| Title | National suicide prevention law |
| Population of Interest | General |
| Family Strategy(ies) | Yes |
| Prevention | |
| Suicide-related awareness, education, psychoeducation for families | Not reported |
| Increase familial resilience | Not reported |
| Family-based psychosocial counselling | Not reported |
| Other preventions | - Team of care for people at risk of completing suicide should be interdisciplinary and should include family - Medical coverage should be provided to individuals at risk of suicide and the families bereaved by suicide regardless of socioeconomic status |
| Intervention | |
| Acute family-related intervention strategies | Not reported |
| Other interventions | Not reported |
| Postvention | |
| Support for families bereaved by suicide | - National interest to provide support to families of those bereaved by suicide   - Interventions after death by suicide aimed to work with families of the deceased |
| Other postventions | Not reported |
| Guideline’s rationale for including family-based intervention(s) in recommendations | |
| Family as a risk factor | Not reported |
| Family as a protective factor | Not reported |
| Other | Not reported |
| Guideline’s measures of implementation and effectiveness of family-based intervention(s) | |
| Measures of Implementation | - National State must guarantee development of joint actions aimed at implementing the national suicide prevention law |
| Measures of Effectiveness | - Executives must regulate this law within ninety (90) days from its introduction |
| Inclusion of action plan or progress reported | Not reported |
| Rate of suicide from WHO | 9.2 per 100 000 |

| Brazil ^48^ | |
| --- | --- |
| Year | 2017-2020 |
| Title | Agenda of strategic actions for surveillance and prevention of suicide and promotion of health in Brazil |
| Population of Interest | General |
| Family Strategy(ies) | Yes |
| Prevention | |
| Suicide-related awareness, education, psychoeducation for families | - Disseminate health promotion component materials including information on violence prevention and culture of peace, harmful use of drugs and alcohol, importance of emotional and social skills to parents, teachers and family members |
| Increase familial resilience | Not reported |
| Family-based psychosocial counselling | Not reported |
| Other preventions | Not reported |
| Intervention | |
| Acute family-related intervention strategies | - Establish Health Care Networks for detecting risk and monitoring of suicide attempts in families |
| Other interventions | Not reported |
| Postvention | |
| Support for families bereaved by suicide | - Guide health departments to identify and support survivors of a suicide attempt and their families |
| Other postventions | Not reported |
| Guideline’s rationale for including family-based intervention(s) in recommendations | |
| Family as a risk factor | Not reported |
| Family as a protective factor | Not reported |
| Other | Not reported |
| Guideline’s measures of implementation and effectiveness of family-based intervention(s) | |
| Measures of Implementation | - Sharing of responsibilities between Ministry of Health, Secretaries of State, Municipal Health, and other government sectors to implement objectives and establish deadlines - Engage local pilot projects to implement the operation of the National Prevention Guidelines of Suicide |
| Measures of Effectiveness | - Strengthen the surveillance of health care by promoting the effective and timely use of data from information systems - Foster partnerships with educational institutions and research, professionals and civil society to develop studies that evaluate the effectiveness of interventions |
| Inclusion of action plan or progress reported | - This guideline itself is a strategic action plan that outlines implementation steps |
| Rate of suicide from WHO | 6.5 per 100 000 |
| Chile ^64^ | |
| Year | 2013 |
| Title | National program for the prevention of suicide: Guidelines for implementation |
| Population of Interest | General |
| Family Strategy(ies) | Yes |
| Prevention | |
| Suicide-related awareness, education, psychoeducation for families | Not reported |
| Increase familial resilience | Not reported |
| Family-based psychosocial counselling | Not reported |
| Other preventions | Not reported |
| Intervention | |
| Acute family-related intervention strategies | - Practical guides developed to detect suicide risk in:   - Children: Items include identification of parenting difficulties, family history of suicide, familial conflict   - Adolescents: Items include familial mental illness, familial conflict, family history of suicidal behaviour   - Adults: Items include family fear of suicide attempt, family history of suicidal behaviour   - Elderly: family history of suicidal behaviour - Emergency psychological help and crisis intervention includes telling the person at risk of dying by suicide that family and friends care about them - Suicide risk management in primary care states that if suicidal ideation is not detected, the family should be told to be attentive to mood changes, negativism, and hopelessness both at beginning of treatment and when stress levels are high |
| Other interventions | Not reported |
| Postvention | |
| Support for families bereaved by suicide | - Families bereaved by suicide will be enrolled in preventive and therapeutic programs, consisting of at least 6 annual interventions including: family follow-ups with health professionals, individual, family or group psychosocial interventions, educational programs to detect risk factors etc. |
| Other postventions | Not reported |
| Guideline’s rationale for including family-based intervention(s) in recommendations | |
| Family as a risk factor | - Family history of suicide increases risk of suicide by 8 times   - Risk factors for suicide in children include a chaotic emotional climate in the family, mental illness in family, familial abuse, death of loved ones, perception of goals as unachievable   - Risk factors for suicide in adults with depression include cases of self-destruction in the family and familial disorder   - Risk factors for suicide in elderly include loss off loved ones, widowhood, unwanted migration or “ping-pong” between households, admission into nursing homes |
| Family as a protective factor | Not reported |
| Other | - Suicidal pacts and family suicides are prevalent in countries India, China, and Sri Lanka - Family members bereaved by suicide may feel guilt and remorse, among other emotions that increase their risk of suicide   - Children bereaved by suicide often feel abandoned, lonely, confused, fearful, angry, and may become emotionally numb |
| Guideline’s measures of implementation and effectiveness of family-based intervention(s) | |
| Measures of Implementation | - Practical guides, preventative and therapeutic programs can be implemented by mental health specialists, clinicians, health technicians, and general community agents - Regional teams will monitor the incorporation of family members bereaved by suicide in preventative and therapeutic programs |
| Measures of Effectiveness | - Epidemiological information (e.g. family structure) collected on a case study basis |
| Inclusion of action plan or progress reported | Not reported |
| Rate of suicide from WHO | 10.6 per 100 000 |
| Guyana^67^ | |
| Year | 2015-2020 |
| Title | A National suicide prevention strategy for Guyana |
| Population of Interest | General |
| Family Strategy(ies) | Yes |
| Prevention | |
| Suicide-related awareness, education, psychoeducation for families | - Provide information and support for families concerned about someone who may be at risk of suicide |
| Increase familial resilience | Not reported |
| Family-based psychosocial counselling | - Individuals being cared for by mental health professionals, primary care or social services are encouraged to involve family in care |
| Other preventions | Not reported |
| Intervention | |
| Acute family-related intervention strategies | Not reported |
| Other interventions | Not reported |
| Postvention | |
| Support for families bereaved by suicide | - Emphasize vigilance of clinicians to the vulnerability of family members bereaved by suicide - Local response teams to support families bereaved by suicide, who can attend to aftermath of a suicide and provide emotional aid |
| Other postventions | Not reported |
| Guideline’s rationale for including family-based intervention(s) in recommendations | |
| Family as a risk factor | - Risk factors for suicide include cultural and familial influences, familial violence, those bereaved by suicide |
| Family as a protective factor | Not reported |
| Other | - Statistics from Guyana   - Case studies showed that family discord (31%), partner relationship problems (25%), domestic violence (11 %) and interpersonal conflict (11%), together accounted for 60% of suicide attempts   - Family dysfunction was found to be a risk factor 34.5% of the time - Less common risk factors in case study found to be family history of suicide |
| Guideline’s measures of implementation and effectiveness of family-based intervention(s) | |
| Measures of Implementation | - Develop protocols that guide mental health services when someone dies by suicide in their care; protocols may include information on preparing for inquests and dealing with the family, including the impact of the suicide and the inquest on the family - Prepare a handbook as a guide for bereaved families and friends - Develop a care plan for professionals who have overviews of specific cases and are responsible for answering any questions from the patient or family - Statistical reports and unit supervision used to verify the utilization of strategies |
| Measures of Effectiveness | - Monitoring incidence and prevalence of suicidal behaviour in country’s 10 regions, by identifying and recording the number of attempted and completed suicide - Review documents and procedures collected in all levels of the surveillance system to assess:   - management and treatment of suicidal behaviour in all levels of healthcare (emergency, inpatient and outpatient services, mental health services, communities)   - quality and effectiveness of proposed interventions |
| Inclusion of action plan or progress reported | Not reported |
| Rate of suicide from WHO | 29.2 per 100 000 |
| Suriname^49^ | |
| Year | 2016-2020 |
| Title | National suicide prevention and intervention plan |
| Population of Interest | General |
| Family Strategy(ies) | Yes |
| Prevention | |
| Suicide-related awareness, education, psychoeducation for families | - Identify vulnerable groups of parents unaware of how to cope with children at risk of suicide and provide resources |
| Increase familial resilience | Not reported |
| Family-based psychosocial counselling | Not reported |
| Other preventions | Not reported |
| Intervention | |
| Acute family-related intervention strategies | Not reported |
| Other interventions | Not reported |
| Postvention | |
| Support for families bereaved by suicide | - Family members bereaved by suicide should receive guidance and support   - System where family members are connected immediately after a suicide to support and guidance for the grieving process   - Grief counselling groups and support groups established in the community for family members bereaved by suicide     - Training volunteers to help administer support groups for families, and enforcing a system that allows them to intervene when necessary   - Website for information and support of fellow people bereaved by suicide created through social media |
| Other postventions | Not reported |
| Guideline’s rationale for including family-based intervention(s) in recommendations | |
| Family as a risk factor | - Surinamese have strong familial ties but lack skills in problem solving and non-violent conflict resolution which may result in familial suicide - Surinamese of the Hindustani descent are at risk of higher levels of stress due to forced choice partners and views on family honour - Among LGBTI severe cases of suicide and long-lasting depression is fueled by factors such as lack of familial support - Family psychopathology, family history of suicidal behaviour and neglect have been identified as risk factors but no data exists on these risk factors in Suriname |
| Family as a protective factor | - Protective factors listed as strong positive personal relationships and support from family - Inventory of best practices used at local, regional, and national levels |
| Other | Not reported |
| Guideline’s measures of implementation and effectiveness of family-based intervention(s) | |
| Measures of Implementation | - Collaboration between different sectors of government - Introduce a reporting requirement for suicidality |
| Measures of Effectiveness | - Central database of past, current and present investigations, plans, and projects, will be established - Task force that draws up national research agenda information related to suicide prevention - Expansion of department dealing with collection and monitoring of suicide related data |
| Inclusion of action plan or progress reported | - The document itself includes and action plan for the implementation of listed strategies |
| Rate of suicide from WHO | 22.8 per 100 000 |
| Uruguay ^39^ | |
| Year | 2011-2015 |
| Title | National Suicide Prevention Plan, For Uruguay 2011-2015 "A commitment to life" |
| Population of Interest | General |
| Family Strategy(ies) | Yes |
| Prevention | |
| Suicide-related awareness, education, psychoeducation for families | - Design a communication strategy to raise awareness about suicide risk for all level of users, including families, community and mass media. |
| Increase familial resilience | Not reported |
| Family-based psychosocial counselling | Not reported |
| Other preventions | - Design interdisciplinary teams that ensure comprehensive attention is given to people at risk and their family nucleus - Expand the coverage of mental health benefits to include the family group as well as the person at risk |
| Intervention | |
| Acute family-related intervention strategies | - Create protocols for the organization of care and monitoring of suicide risk for both the person and family members, at different levels of care (clinics, Health Centers, Urgent and Emergency Services etc.) |
| Other interventions | Not reported |
| Postvention | |
| Support for families bereaved by suicide | Not reported |
| Other postventions | Not reported |
| Guideline’s rationale for including family-based intervention(s) in recommendations | |
| Family as a risk factor | Not reported |
| Family as a protective factor | Strong family ties listed as a protective factor for suicide |
| Other | Not reported |
| Guideline’s measures of implementation and effectiveness of family-based intervention(s) | |
| Measures of Implementation | - The National Mental Health Program will be in charge of the monitoring of the implementation of interventions for people at risk and family members |
| Measures of Effectiveness | - Evaluation indicators assess the level of providers that have been included and how many of them comply with the interventions mentioned in the plan - National Commission of National Plan for the Prevention of Suicide will be responsible to articulate, supervise, evaluate and monitor the plan |
| Inclusion of action plan or progress reported | - National day of suicide prevention in 2019 allowed for a summary of progress and amendments made using the national strategy^39^ |
| Rate of suicide from WHO | 18.4 per 100 000 |
| Supplemental table 7.0 Guidelines identified from North America | |
| Canada^23^ | |
| Year | 2018 |
| Title | The Federal Framework for Suicide Prevention-Progress Report 2018 |
| Population of Interest | General |
| Family Strategy(ies) | Yes |
| Prevention | |
| Suicide-related awareness, education, psychoeducation for families | - Listening to One Another to Grow Strong: Culturally-Based, Family Centered Mental Health Promotion for Indigenous Youth - The Public Health Agency help Canadian Coalition for Seniors Mental Health and Shoppers Drugmart collaborate to develop resources for seniors and their families to increase awareness of mental health and depression in seniors - Health Canada partnered with LifeSpeak, a video library for families that showcases health and wellness information on topics including suicide - A Veterans Emergency Fund, the Veteran Family Program, new Education and Training Benefit and online support for Veteran families and caregivers |
| Increase familial resilience | - The First Nations Mental Wellness Continuum Framework is a recourse for partners looking to work with the community and focuses on interconnectedness between life purpose, hope, and a sense of belonging within families, communities and culture |
| Family-based psychosocial counselling | Not reported |
| Other preventions | Not reported |
| Intervention | |
| Acute family-related intervention strategies | Not reported |
| Other interventions | Not reported |
| Postvention | |
| Support for families bereaved by suicide | Not reported |
| Other postventions | Not reported |
| Guideline’s rationale for including family-based intervention(s) in recommendations | |
| Family as a risk factor | Not reported |
| Family as a protective factor | Not reported |
| Other | Not reported |
| Guideline’s measures of implementation and effectiveness of family-based intervention(s) | |
| Measures of Implementation | - $1.5 billion over five years for First Nations and Inuit families to help with effectiveness of treatment and prevention services in communities with high need |
| Measures of Effectiveness | - Public Health Agency of Canada published the Suicide Surveillance Indicator Framework, which has indicators for risk and protective factors at the individual, family, community and societal level |
| Inclusion of action plan or progress reported | - In 2019 the Government of Canada published a contextual analysis of the Suicide Surveillance Indicators, including assessment of family level risk and protective factors (family history of suicide, family relationship, family mental health and substance use)^27^ |
| Rate of suicide from WHO | 12.5 per 100 000 |
| Canada^23^ | |
| Year | 2016 |
| Title | National Inuit Suicide Prevention Strategy |
| Population of Interest | Inuit population |
| Family Strategy(ies) | Yes |
| Prevention | |
| Suicide-related awareness, education, psychoeducation for families | - Healthcare providers must involve family and community in prevention and treatment of Inuit for suicide risk to ensure that skills are transferred to the community and cultural context is kept intact   - Thus, mental health workers should have training and knowledge of the Inuit community |
| Increase familial resilience | - Reduction of risk by building resilience in individuals, families, and communities   - Through the implementation of strategy organizations will work with Inuit regions to ensure that especially vulnerable children and families are safe, and to reduce intergenerational trauma by providing family strengthening resources |
| Family-based psychosocial counselling | Not reported |
| Other preventions | Support healthy development of children by supporting families to create cohesive, non-violent spaces for growth |
| Intervention | |
| Acute family-related intervention strategies | Not reported |
| Other interventions | Not reported |
| Postvention | |
| Support for families bereaved by suicide | Not reported |
| Other postventions | Not reported |
| Guideline’s rationale for including family-based intervention(s) in recommendations | |
| Family as a risk factor | - Stress, prejudice, social inequity, social challenges such as poverty and poor health status faced by the Inuit community led to higher risk of suicide - The Adverse Childhood Experiences (ACE) study, shows that childhood abuse, neglect and family dysfunction negatively shapes lifelong outcomes and can increase risk of suicide |
| Family as a protective factor | Not reported |
| Other | - Religious missionaries led by the Royal Canadian Mountain Police coerced people living in family units to live in settlements that were promised to be free but were not due to the withholding of a family allowance - Canadian residential schools were imposed onto families and children |
| Guideline’s measures of implementation and effectiveness of family-based intervention(s) | |
| Measures of Implementation | - National, local and community level collaboration and partnerships allow the strategy to be implemented and to reach all Inuit population |
| Measures of Effectiveness | - The strategy outlines how different stakeholders can coordinate with each other more effectively to mitigate suicide risk |
| Inclusion of action plan or progress reported | Not reported |
| Rate of suicide from WHO | 12.5 per 100 000 |
| Canada^23^ | |
| Year | 2017 |
| Title | Joint suicide prevention strategy |
| Population of Interest | Canadian armed forces (CAF) and veteran affairs Canada (VAC) |
| Family Strategy(ies) | Yes |
| Prevention | |
| Suicide-related awareness, education, psychoeducation for families | - The CAF provides comprehensive education, training, mental and spiritual health programs, rehabilitation programs, transition services, and extensive support for families |
| Increase familial resilience | Not reported |
| Family-based psychosocial counselling | - Services provide veterans and families 24/7 confidential access to mental health professionals and up to 20 sessions of counselling free of charge |
| Other preventions | - The Operational Stress Injury Social Support (OSISS) program offers confidential peer support to CAF members, veterans and their families through trained peer support and family peer support coordinators - VAC provides funding for family caregiver relief benefits |
| Intervention | |
| Acute family-related intervention strategies | Not reported |
| Other interventions | Not reported |
| Postvention | |
| Support for families bereaved by suicide | - Caring Contacts mental health crisis protocol provides for bereaved & grieving families |
| Other postventions | Not reported |
| Guideline’s rationale for including family-based intervention(s) in recommendations | |
| Family as a risk factor | - Veteran by suicide has a ripple effect on families, friends, and communities - Relationship conflict, suicide by family member, are risk factors; whereas family connections and positive social interaction is a protective factor |
| Family as a protective factor | Not reported |
| Other | Not reported |
| Guideline’s measures of implementation and effectiveness of family-based intervention(s) | |
| Measures of Implementation | Not reported |
| Measures of Effectiveness | - VAC is investing in a Veteran and Family Well-Being Fund, that will promote innovative research and services |
| Inclusion of action plan or progress reported | Not reported |
| Rate of suicide from WHO | 12.5 per 100 000 |
| Canada ^23^ | |
| Year | 2013 |
| Title | National Aboriginal Youth Suicide Prevention Strategy (NA YSPS) |
| Population of Interest | Between ages 10-30; First Nations youth living on a reserve; and,  Inuit youth living in an Inuit community |
| Family Strategy(ies) | Yes |
| Prevention | |
| Suicide-related awareness, education, psychoeducation for families | - Improved access to quality programs and services, and education for Aboriginal youth and families |
| Increase familial resilience | Not reported |
| Family-based psychosocial counselling | Not reported |
| Other preventions | - Promote Aboriginal youth, families, and communities taking part in projects, activities, and services that prevent suicide - Activities for youth that increase their connection to community and promote cultural continuity, the land, each other, Elders, their family |
| Intervention | |
| Acute family-related intervention strategies | Not reported |
| Other interventions | Not reported |
| Postvention | |
| Support for families bereaved by suicide | Not reported |
| Other postventions | Not reported |
| Guideline’s rationale for including family-based intervention(s) in recommendations | |
| Family as a risk factor | - Youth experiences are rooted in cultural disintegration, breakdown of family structures - Previous attempts, a family history or community ‘legacy’ of suicide are risk factors for suicide; family attention, support and care are protective factors |
| Family as a protective factor | Not reported |
| Other | Not reported |
| Guideline’s measures of implementation and effectiveness of family-based intervention(s) | |
| Measures of Implementation | - Inuit-specific strategy activities are guided by a Implementation Guide that was designed by Inuit Tapiriit Kanatami’s (ITK), Health Canada Regional offices, and ITK’s National Inuit Youth Council |
| Measures of Effectiveness | Not reported |
| Inclusion of action plan or progress reported | Not reported |
| Rate of suicide from WHO | 12.5 per 100 000 |
| Costa Rica ^61^ | |
| Year | 2010 |
| Title | File No. 17.847 - Creation of the National Institute of Suicide Prevention |
| Population of Interest | General |
| Family Strategy(ies) | Yes |
| Prevention | |
| Suicide-related awareness, education, psychoeducation for families | Not reported |
| Increase familial resilience | Not reported |
| Family-based psychosocial counselling | Duties and powers of Board of Directors include:   - Creation and training of associations made up of individuals and families facing this phenomenon - Execute preventative workshops that address the person and families dealing with suicide related issues - Create a permanent support group for individuals and families who need urgent attention |
| Other preventions | Not reported |
| Intervention | |
| Acute family-related intervention strategies | Not reported |
| Other interventions | Not reported |
| Postvention | |
| Support for families bereaved by suicide | Not reported |
| Other postventions | Not reported |
| Guideline’s rationale for including family-based intervention(s) in recommendations | |
| Family as a risk factor | - Familial conflict and death of a family member identified as risks for suicide |
| Family as a protective factor | Not reported |
| Other | Not reported |
| Guideline’s measures of implementation and effectiveness of family-based intervention(s) | |
| Measures of Implementation | - Representatives from Ministry of Health, Ministry of Public Education, National Institute for Women, National Children’s Trust, Institute on Alcohol and Drug Dependence, National Council for Rehabilitation, civil society, Criminological Institute, Costa Rican Social Security, will be elected to a Board of Directors who will carry out family-based duties and goals listed in guideline - The legislative assembly has the obligation to hold public institutions, civil society organizations and district councils accountable on program/project compliance during March of each year |
| Measures of Effectiveness | - The Chief Executive Officer of the Board of Directors will carry out audits or special studies related to the duties of the National Institute for Suicide Prevention |
| Inclusion of action plan or progress reported | Not reported |
| Rate of suicide from WHO | 7.9 per 100 000 |
| Dominican Republic^58^ | |
| Year | 2014 |
| Title | Prevention program in suicidal behavior the Dominican Republic |
| Population of Interest | General |
| Family Strategy(ies) | Yes |
| Prevention | |
| Suicide-related awareness, education, psychoeducation for families | - Educational goals: Development of training programs about discipline and good use of free time in teenagers, and appropriate parenting patterns - Health sector goals: training for community leaders in promoting protective factors such as positive family patterns (good relationships with members, familial support) and psychoeducation to relatives of people who tried suicide |
| Increase familial resilience | Not reported |
| Family-based psychosocial counselling | Not reported |
| Other preventions | - Labour goals: Implement strategies and workshops to strengthen teamwork and interpersonal skills - Penitentiary goals: Psychoeducational tool development to help mitigate socialization problems and creation of self-help groups |
| Intervention | |
| Acute family-related intervention strategies | Not reported |
| Other interventions | Not reported |
| Postvention | |
| Support for families bereaved by suicide | Not reported |
| Other postventions | Not reported |
| Guideline’s rationale for including family-based intervention(s) in recommendations | |
| Family as a risk factor | Not reported |
| Family as a protective factor | - Close relationships and family as protective factors for suicide and poor parenting and family conflict as risk factors |
| Other | Not reported |
| Guideline’s measures of implementation and effectiveness of family-based intervention(s) | |
| Measures of Implementation | - Close relationships and family as protective factors for suicide and poor parenting and family conflict as risk factors |
| Measures of Effectiveness | - Strengthen epidemiological surveillance system by providing information related to suicide   - Work to increase the number of health centers and epidemiological newsletters that report suicide related data - Promote research and collection of evidence related to suicide   - Work to increase number of universities that conduct research on suicide - Evaluation of short-term goals: quarterly monitoring and evaluation of interventions according to the Annual Operating Plan (AOP) - Evaluation of long-term goals: Use of indicators such as suicide rate per 100 000 people, suicide attempt rate per 100 000, level of attention (service utilization), monitoring of suicides in primary care (referrals and follow-ups completed in services used), and calculation of life expectancy adjusted for disability, to evaluate long-term strategies |
| Inclusion of action plan or progress reported | Not reported |
| Rate of suicide from WHO | 9.9 per 100 000 |
| El Salvador ^69^ | |
| Year | 2018 |
| Title | Technical guidelines for the promotion of mental health, prevention, early identification and approach to suicidal behavior in adolescents and young women with an emphasis on pregnancy |
| Population of Interest | Adolescents and young pregnant women |
| Family Strategy(ies) | Yes |
| Prevention | |
| Suicide-related awareness, education, psychoeducation for families | - Young pregnant women interventions focus on:   - Teaching new fathers the importance of Community Family Health Units and provide the family with resources to combat alcohol use - Adolescent interventions focus on:   - helping parents and family to understand depression, and provide support by teaching active listening |
| Increase familial resilience | Not reported |
| Family-based psychosocial counselling | - Adolescent interventions focus on:   - Provide family counseling to improve communication.   - Schedule home visits by responsible health personnel, for support and follow-up of the adolescents and families - Young pregnant women interventions focus on:   - Group counselling for families and individual   - Health personnel guide family members to strengthen or re-establish their network of psychosocial support |

| Other preventions | Not reported |
| --- | --- |
| Intervention | |
| Acute family-related intervention strategies | Not reported |
| Other interventions | Not reported |
| Postvention | |
| Support for families bereaved by suicide | Not reported |
| Other postventions | Not reported |
| Guideline’s rationale for including family-based intervention(s) in recommendations | |
| Family as a risk factor | - Psychosocial risk factors for adolescents include family background involving violence, psychological problems in family, substance abuse in family - Women who have experienced gender based violence in families, sexual abuse in families, and unwanted pregnancies due to familial pressure can lead to risk of suicide |
| Family as a protective factor | Not reported |
| Other | Not reported |
| Guideline’s measures of implementation and effectiveness of family-based intervention(s) | |
| Measures of Implementation | - Ministry of Health responsible for carrying out intervention plans |
| Measures of Effectiveness | - Ministry of Health will organize and develop research efforts for psychological problems in pregnant teens and adolescents |
| Inclusion of action plan or progress reported | Not reported |
| Rate of suicide from WHO | 13.7 per 100 000 |
| Nicaragua^72^ | |
| Year | 2000 |
| Title | National suicide prevention strategy national plan for the promotion of life |
| Population of Interest | General |
| Family Strategy(ies) | Yes |
| Prevention | |
| Suicide-related awareness, education, psychoeducation for families | Not reported |
| Increase familial resilience | Not reported |
| Family-based psychosocial counselling | Not reported |
| Other preventions | Not reported |
| Intervention | |
| Acute family-related intervention strategies | - Family members should take all threats seriously, be attentive to mood changes and persistent hopelessness in family members, focus attention on members who have suffered traumatic events - Hospitalization of person at risk of suicidal behaviour, brings the family together and allows them to see a comprehensive picture of the person’s suffering - If acute suicide risk is present then family should be involved at intake, they will be asked about individual, and they will be informed about the seriousness of the issue - If acute risk is dissolved and the individual is unwilling to return to family, home visits will be completed by health personnel - If individual is medicated, dose will be handed to family - Psychiatric evaluation should be completed immediately once risk is identified to minimize the chance that family will withhold information on suicide related occurrences - In adolescent hospitalization family members should be told the diagnosis; once discharge from hospital, health personnel observe both family and individual for suicide risk |
| Other interventions | Not reported |
| Postvention | |
| Support for families bereaved by suicide | - For those bereaved by suicide, they are told to overcome familial perceived failure and shame by recovering self-esteem |
| Other postventions | Not reported |
| Guideline’s rationale for including family-based intervention(s) in recommendations | |
| Family as a risk factor | - Family dysfunction listed as a risk factor for suicide - Family history of suicides, self-harm, abuse, alcohol or drug use in parents, and early death of parents, lack of support from family identified as risk factors for suicide |
| Family as a protective factor | Not reported |
| Other | - Cultural stigma of suicides leads families to hide suicides that have occurred |
| Guideline’s measures of implementation and effectiveness of family-based intervention(s) | |
| Measures of Implementation | - Healthcare personnel should trained to encourage the natural leaders in the community to become health promoters who can help families gain interest in therapeutic interventions |
| Measures of Effectiveness | - Organization of multi-sectoral and national activities to increase knowledge about suicidal behavior and its effective prevention |
| Inclusion of action plan or progress reported | Not reported |
| Rate of suicide from WHO | 12.2 per 100 000 |
| Panama^26^ | |
| Year | 2006 |
| Title | Plan for the prevention and control of suicidal behavior in panama |
| Population of Interest | General |
| Family Strategy(ies) | Yes |
| Prevention | |
| Suicide-related awareness, education, psychoeducation for families | Not reported |
| Increase familial resilience | Not reported |
| Family-based psychosocial counselling | Not reported |
| Other preventions | - Policy attention given to the social rights of people and families vulnerable to suicide |
| Intervention | |
| Acute family-related intervention strategies | Not reported |
| Other interventions | Not reported |
| Postvention | |
| Support for families bereaved by suicide | Not reported |
| Other postventions | Not reported |
| Guideline’s rationale for including family-based intervention(s) in recommendations | |
| Family as a risk factor | Not reported |
| Family as a protective factor | Not reported |
| Other | - Suicide significantly reduces years of productive life for both the individual and their families |
| Guideline’s measures of implementation and effectiveness of family-based intervention(s) | |
| Measures of Implementation | - Collaborative sub-commission that reviews policies, laws and regulations on suicide and proposes implementation plans |
| Measures of Effectiveness | Not reported |
| Inclusion of action plan or progress reported | Not reported |
| Rate of suicide from WHO | 4.3 per 100 000 |
| United States of America ^60^ | |
| Year | 2012 |
| Title | National Strategy for Suicide Prevention: Goals and Objectives for Action - A report of the U.S. Surgeon General and of the National Action Alliance for Suicide Prevention |
| Population of Interest | General |
| Family Strategy(ies) | Yes |
| Prevention | |
| Suicide-related awareness, education, psychoeducation for families | - The first strategic direction “Healthy and Empowered Individuals, Families, and Communities” discusses:   - Integrate suicide prevention into the values, culture, leadership, and work of family organizations   - Increase family members’ knowledge of the warning signs for suicide and of how to connect to individuals in crisis with assistance and care   - Promote effective programs based on family connectedness that increase protection from suicide risk in isolated and marginalized groups   - Health care systems and clinicians can promote the understanding to family members that recovery from mental disorders is real and possible to prevent stigma and to establish family as a potential source of support |
| Increase familial resilience | Not reported |
| Family-based psychosocial counselling | Not reported |
| Other preventions | Not reported |
| Intervention | |
| Acute family-related intervention strategies | - Strategic direction 2 “Clinical and community preventative services” includes family based objectives as well:   - Families should store and lock household firearms and ammunition separately, dispose of unwanted medications, take extra precautions if a member of the family is at high risk   - Families should learn the signs and symptoms of suicidal behaviour and how to connect to people at risk |
| Other interventions | Not reported |
| Postvention | |
| Support for families bereaved by suicide | Postvention   - Compassionate care for those bereaved by suicide (including family members) from first responders and access to a variety of support resources during grieving process (e.g. self-help literature, survivor outreach teams, in person and online support groups, clinical referral systems who specialize in grief |
| Other postventions | - Strategic direction 3 “Treatment and Support Services” includes family based objectives as well:   - Families should be coached on the importance of continuity of care post-discharge   - Family members of recently discharged individuals can learn when to contact emergency services for loved ones, follow-up with appointment reminders and treatment plans   - Provide clinical care such as trauma treatment for families of individuals who have attempted or died by suicide |
| Guideline’s rationale for including family-based intervention(s) in recommendations | |
| Family as a risk factor | Not reported |
| Family as a protective factor | - Greater social support and larger families may protect some groups from suicide - Family connectedness has been found to play a strong protective role against suicide for the lesbian, gay, bisexual youth |
| Other | - United Kingdom study showed that sharing information with families after a suicide contributed to reduction in suicide rates seen in the country in 2012. |
| Guideline’s measures of implementation and effectiveness of family-based intervention(s) | |
| Measures of Implementation | - Public and private partnership to guide listed interventions - Coordinated leadership between national, state, local, and tribal levels |
| Measures of Effectiveness | - Strategic Direction 4 “Surveillance, Research, and Evaluation” directs families to:   - Participate in surveys and other data collection efforts addressing suicide   - Support evaluation of suicide prevention programs   - Evaluate the impact and effectiveness of suicide prevention interventions and disseminate findings     - Examine how suicide prevention efforts are implemented in different communities to determine the most effective and efficient method of implementation     - Evaluate the impact and effectiveness of the National Strategy in reducing suicide mortality |
| Inclusion of action plan or progress reported | Not reported |
| Rate of suicide from WHO | 15.3 per 100 000 |
| United States of America^24^ | |
| Year | 2011-2015 |
| Title | National suicide prevention strategic plan |
| Population of Interest | American Indian/ Alaskan Native |
| Family Strategy(ies) | Yes |
| Prevention | |
| Suicide-related awareness, education, psychoeducation for families | Not reported |
| Increase familial resilience | Not reported |
| Family-based psychosocial counselling | Not reported |
| Other preventions | - Promotion of communication between families, community members, and organizations to help reduce risk of suicide |
| Intervention | |
| Acute family-related intervention strategies | Not reported |
| Other interventions | Not reported |
| Postvention | |
| Support for families bereaved by suicide | Not reported |
| Other postventions | Not reported |
| Guideline’s rationale for including family-based intervention(s) in recommendations | |
| Family as a risk factor | Not reported |
| Family as a protective factor | Not reported |
| Other | - Social, educational, and cultural issues underlie suicide (e.g. poverty, lack of economic opportunity, limited educational alternatives, community breakdown, familial disruption, and stigma) |
| Guideline’s measures of implementation and effectiveness of family-based intervention(s) | |
| Measures of Implementation | - The suicide prevention council provides recommendations and guidance to the Indian Health System regarding suicide prevention, intervention, and postvention in Indian Country |
| Measures of Effectiveness | Not reported |
| Inclusion of action plan or progress reported | Not reported |
| Rate of suicide from WHO | 15.3 per 100 000 |

Supplemental table 8.0: AGREE II risk of bias scores from two appraisers for 6 domains and guideline overall

|  | Scope and Purpose (%) | Stakeholder Involvement (%) | Rigour of Development (%) | Clarity of Presentation (%) | Applicability (%) | Editorial Independence (%) | Overall Guideline Assessment (%) |
| --- | --- | --- | --- | --- | --- | --- | --- |
| Afghanistan | 50.0 | 27.8 | 20.8 | 44.4 | 16.7 | 41.7 | 50.0 |
| Argentina | 44.4 | 27.8 | 29.7 | 22.2 | 33.3 | 41.7 | 33.3 |
| Australia (not including action plan) | 61.1 | 61.1 | 54.2 | 61.1 | 50.0 | 50.0 | 75.0 |
| Australia - aboriginal | 61.1 | 61.1 | 43.8 | 50.0 | 45.8 | 50 | 66.7 |
| Austria | 44.4 | 33.3 | 25.0 | 33.3 | 16.7 | 41.7 | 50.0 |
| Belarus | 27.8 | 33.3 | 25.0 | 27.8 | 16.7 | 33.3 | 33.3 |
| Belgium | 61.1 | 50.0 | 68.8 | 61.1 | 50.0 | 50.0 | 75.0 |
| Bhutan | 55.6 | 50.0 | 50.0 | 44.4 | 58.3 | 41.7 | 75.0 |
| Brazil | 55.6 | 44.4 | 54.2 | 38.9 | 58.3 | 41.7 | 66.7 |
| Bulgaria (not including action plan) | 44.4 | 33.3 | 33.3 | 50.0 | 29.2 | 41.7 | 50.0 |
| Canada - aboriginal | 44.4 | 55. 6 | 45.8 | 50.0 | 45.8 | 50.0 | 66.7 |
| Canada - aboriginal youth | 55.6 | 55.6 | 41.7 | 50.0 | 45.8 | 50.0 | 66.7 |
| Canada -progress report (not including implementation plan) | 55.6 | 55.6 | 41.7 | 55.6 | 45.8 | 50 | 66.7 |
| Canada - veteran | 50.0 | 50 | 43.75 | 50 | 45.8 | 50 | 66.7 |
| Chile | 55.6 | 50 | 54.2 | 50 | 45.8 | 50 | 50 |
| Cook’s island | 38.9 | 38.9 | 31.25 | 50 | 25 | 33.3 | 33.3 |
| Costa Rica | 50.0 | 50 | 50 | 50 | 45.8 | 50 | 50.0 |
| Croatia | 50.0 | 33.3 | 43.75 | 50 | 45.8 | 41.7 | 66.7 |
| Denmark | 50.0 | 44.4 | 35.4 | 50 | 54.2 | 41.7 | 58.3 |
| Dominican Republic | 61.1 | 50 | 56.25 | 50 | 45.8 | 58.3 | 66.7 |
| El Salvador | 61.1 | 50 | 56.25 | 50 | 45.8 | 50 | 66.7 |
| England (Not including action plan) | 50.0 | 44.4 | 45.8 | 50 | 54.2 | 41.7 | 66.7 |
| Fiji | 44.4 | 44.4 | 35.4 | 50 | 33.3 | 33.3 | 33.3 |
| Finland | 50 | 38. 9 | 47.9 | 50 | 45.8 | 50 | 50 |
| France | 44.4 | 44.4 | 37.5 | 44.4 | 50 | 41.7 | 50 |
| Guyana | 55.6 | 44.4 | 41. 7 | 50 | 45.8 | 41.7 | 50 |
| Ireland | 55.6 | 50 | 52.1 | 44.4 | 58.3 | 41.7 | 83.3 |
| Italy – prison population | 50.0 | 44.4 | 33.3 | 50 | 41.7 | 33.3 | 50 |
| Italy – child penitentiary | 50.0 | 44.4 | 33.3 | 50 | 41.7 | 33.3 | 50 |
| Japan (not including published law on suicide) | 50.0 | 27.8 | 33.3 | 50 | 33.3 | 41.7 | 66.7 |
| Lithuania | 50.0 | 44.4 | 37.5 | 44.4 | 33.3 | 33.3 | 50.0 |
| Luxembourg | 44.4 | 44.4 | 39.6 | 44.4 | 33.3 | 33.3 | 50.0 |
| Malaysia | 55.6 | 44.4 | 47.9 | 44.4 | 58.3 | 41.7 | 66.7 |
| Namibia | 55.6 | 55.6 | 45.8 | 50.0 | 45.8 | 50.0 | 50.0 |
| Netherlands (not including prevention agenda) | 55.6 | 50.0 | 41.7 | 44.4 | 41.7 | 33.3 | 50.0 |
| New Zealand | 55.6 | 61.1 | 52.1 | 50.0 | 45.8 | 50.0 | 66.7 |
| Nicaragua | 44.4 | 44.4 | 37.5 | 44.4 | 33.3 | 33.3 | 50.0 |
| Northern Ireland | 55.6 | 55. 6 | 54.2 | 55.6 | 62.5 | 41.7 | 83.3 |
| Norway | 44.4 | 44.4 | 45.8 | 50.0 | 45.8 | 50.0 | 50.0 |
| Panama | 44.4 | 44.4 | 18.8 | 33.3 | 33.3 | 33.3 | 33.3 |
| Portugal | 61.1 | 44.4 | 45.8 | 50.0 | 58.3 | 33.3 | 75.0 |
| Scotland (not including action plan) | 50.0 | 38.9 | 29.7 | 27.8 | 62.5 | 33.3 | 41.7 |
| South Korea (not including act) | 50.0 | 33.3 | 31.25 | 50.0 | 29.2 | 41.7 | 66.7 |
| Spain | 50.0 | 44.4 | 45.8 | 44.4 | 20.8 | 33.3 | 50 |
| Sri Lanka | 44.4 | 27.8 | 6.25 | 22.2 | 12.5 | 16.7 | 16.7 |
| Suriname | 55.6 | 50.0 | 45.8 | 50.0 | 58.3 | 41.7 | 66.7 |
| Sweden | 50.0 | 44.4 | 29.2 | 27. 8 | 54.2 | 33.3 | 50.0 |
| Switzerland | 50.0 | 44.4 | 29.2 | 27. 8 | 54.2 | 33.3 | 50.0 |
| Uruguay  (not including progress report) | 50.0 | 50 | 37.5 | 33.3 | 50.0 | 33.3 | 50.0 |
| USA | 55.6 | 61.1 | 37.5 | 50.0 | 45.8 | 50.0 | 66.7 |
| USA - aboriginal | 44.4 | 50.0 | 33.3 | 55.6 | 45.8 | 50.0 | 66.7 |
| Uzbekistan | 50.0 | 50.0 | 39.6 | 50.0 | 45.8 | 41.7 | 50.0 |

Table 9.0 Search strategy used for MEDLINE database

| Database | Search strategy |
| --- | --- |
| MEDLINE | 1. suicide/ or suicidal ideation/ or suicide, attempted/ 2. suicid*.mp. 3. parasuicid*.mp. 4. 1 or 2 or 3 5. exp guideline/ 6. guideline*.mp. 7. exp. Guidelines as Topic/ 8. exp. consensus development conferences as topic/ 9. exp. consensus development conference/ 10. consensus development conference*.mp. 11. consensus statement*.mp. 12. guideline.pt. 13. 5 or 6 or 7 or 8 or 9 or 10 or 11 or 12 14. 4 and 13 15. limit 14 to yr=“1999-Current” |
